# *Arabidopsis* NMD3 Is Required for Nuclear Export of 60S Ribosomal Subunits and Affects Secondary Cell Wall Thickening

**DOI:** 10.1371/journal.pone.0035904

**Published:** 2012-04-27

**Authors:** Mei-Qin Chen, Ai-Hong Zhang, Quan Zhang, Bao-Cai Zhang, Jie Nan, Xia Li, Na Liu, Hong Qu, Cong-Ming Lu, Sudmorgen, Yi-Hua Zhou, Zhi-Hong Xu, Shu-Nong Bai

**Affiliations:** 1 PKU-Yale Joint Research Center of Agricultural and Plant Molecular Biology, State Key Laboratory of Protein and Plant Gene Research, College of Life Sciences, Peking University and The National Center of Plant Gene Research, Beijing, China; 2 Institute of Botany, Chinese Academy of Sciences, Beijing, China; 3 College of Life Sciences, Peking University, Beijing, China; 4 State Key Laboratory of Plant Genomics, Institute of Genetics and Developmental Biology, Chinese Academy of Sciences, Beijing, China; Lawrence Berkeley National Laboratory, United States of America

## Abstract

NMD3 is required for nuclear export of the 60S ribosomal subunit in yeast and vertebrate cells, but no corresponding function of NMD3 has been reported in plants. Here we report that *Arabidopsis thaliana* NMD3 (AtNMD3) showed a similar function in the nuclear export of the 60S ribosomal subunit. Interference with AtNMD3 function by overexpressing a truncated dominant negative form of the protein lacking the nuclear export signal sequence caused retainment of the 60S ribosomal subunits in the nuclei. More interestingly, the transgenic *Arabidopsis* with dominant negative interference of AtNMD3 function showed a striking failure of secondary cell wall thickening, consistent with the altered expression of related genes and composition of cell wall components. Observation of a significant decrease of rough endoplasmic reticulum (RER) in the differentiating interfascicular fiber cells of the transgenic plant stems suggested a link between the defective nuclear export of 60S ribosomal subunits and the abnormal formation of the secondary cell wall. These findings not only clarified the evolutionary conservation of NMD3 functions in the nuclear export of 60S ribosomal subunits in yeast, animals and plants, but also revealed a new facet of the regulatory mechanism underlying secondary cell wall thickening in *Arabidopsis*. This new facet is that the nuclear export of 60S ribosomal subunits and the formation of RER may play regulatory roles in coordinating protein synthesis in cytoplasm and transcription in nuclei.

## Introduction

Ribosomes have been long known as the main components of the protein synthesis machinery in cells. However, rapidly accumulating evidences suggest regulatory roles of the ribosome in animal development and other biological processes such as diseases, not only through regulation of protein synthesis, but also through extraribosomal functions of ribosomal proteins and ribosomal biogenesis [1]–[4]. Recently, Kondrashov et al. reported ribosome mediates specificity in Hox mRNA translation and vertebrate tissue patterning [5]. In plants, mutations in ribosomal protein genes affecting aspects of development such as embryogenesis (*RPS11A*, *RPL3A*, *RPL8A*, *RPL19A*, *RPL23C*, and *RPL40B*) [6] and leaf shape (*RPL4D*, *RPL5A*, *RPL5B*, *RPL7B*, *RPL9C*, *RPL10aB*, *RPL18C*, *RPL23aA*, *RPL23aB*, *RPL24B*, *RPL27a*, *RPL28A*. *RPL38B*, *RPL39C*, and *RPS6A*, *RPS21B*, *RPS24B*, *RPS28B*) [7]–[12]. Some complicated developmental retardations including late flowering, vacuolar trafficking, and UV response (*RPS3B*, *RPS18A*, *RPL24B*, *RPL4* and *RPL10*) have also been reported [13]–[17]. In addition, five genes related to ribosome biogenesis (*OLIGOCELLUA2, AtNUC-L1*, *EBP1, TORMOZ* and *SLOW WALKER1*) have been reported to be involved in plant development in different ways [18]. However, these findings have only begun to reveal the potential mechanisms of ribosome-mediated regulatory functions.

Eukaryotic ribosome biogenesis, mainly based on studies in yeast, is a complicated process that can be roughly described as two connected phases, ribosome assembly and nuclear export of ribosomal subunits [19]. It is known that the factors XPOl/CRM1 and GTPase Ran are required for nuclear export of both 40S and 60S ribosomal subunits to occur, and three different types of nuclear export adaptor/receptors, NMD3, MEX67-MTR2 and ARX1, have been demonstrated to be involved in the nuclear export of the 60S subunit [19]. Considering the conservation of the ribosome in function and structure among eukaryotic cells, the nuclear export mechanism is reasonably expected to be conserved. Indeed, XPO1/CRM1 has been shown to be involved in the nuclear export of the 60S subunit in both animal and plant cells [20], [21].

NMD3 is a nuclear export adaptor in yeast and vertebrate cells, the function of which is evolutionarily conserved [22], [23]. Although there is a sequence annotated as *NMD3* in the *Arabidopsis* database (detailed sequence information see Figure S1), its function has not yet been reported in plants. Therefore, it is of interest to determine whether this annotated *Arabidopsis thaliana NMD3* (*AtNMD3*) sequence would have similar functions as previously characterized homologs and whether it may have regulatory effects in plant development.

The cellulosic cell wall is a distinct structure in the plant kingdom. It is not only a key element determining plant morphogenesis and integration of plants into their environment, but also a focus of energy resource for human use in recent times [24]–[29]. While many genes have been identified during the past two decades which encode enzymes involving synthesis of cell wall components and transcriptional factors involving the regulation of cell wall formation [30]–[34], the regulatory mechanism of cell wall formation remains elusive. In addition to the reported regulatory effects at various levels such as gene expression, enzyme activity and rosette complex assembly, polysome aggregation also has been reported to correlate with secondary cell wall thickening during cotton fiber development [35]. However, no further evidence is available on whether the polysome aggregation causes secondary cell wall thickening.

In our pilot experiments, we found that transient downregulation of the *Arabidopsis* homolog of *NMD3*, *AtNMD3*, resulted in pleiotropic phenotypes in T1 transgenic plants, including a defect in secondary cell wall thickening and failure of endothecium development (Figure S2). These findings suggested that *AtNMD3* may have a regulatory role in plant development. In this study, we first explored the role of AtNMD3 in the nuclear export of ribosome 60S subunits, as has been reported in yeast and vertebrate cells. Additionally, transgenic plants expressing the dominant negative form of *AtNMD3* were examined, revealing a striking phenotype of defective secondary cell wall thickening. Finally, a close correlation between rough endoplasmic reticulum (RER) formation and secondary cell wall thickening was observed.

## Results

### Phylogenetic Analysis of AtNMD3

**Figure 1:**
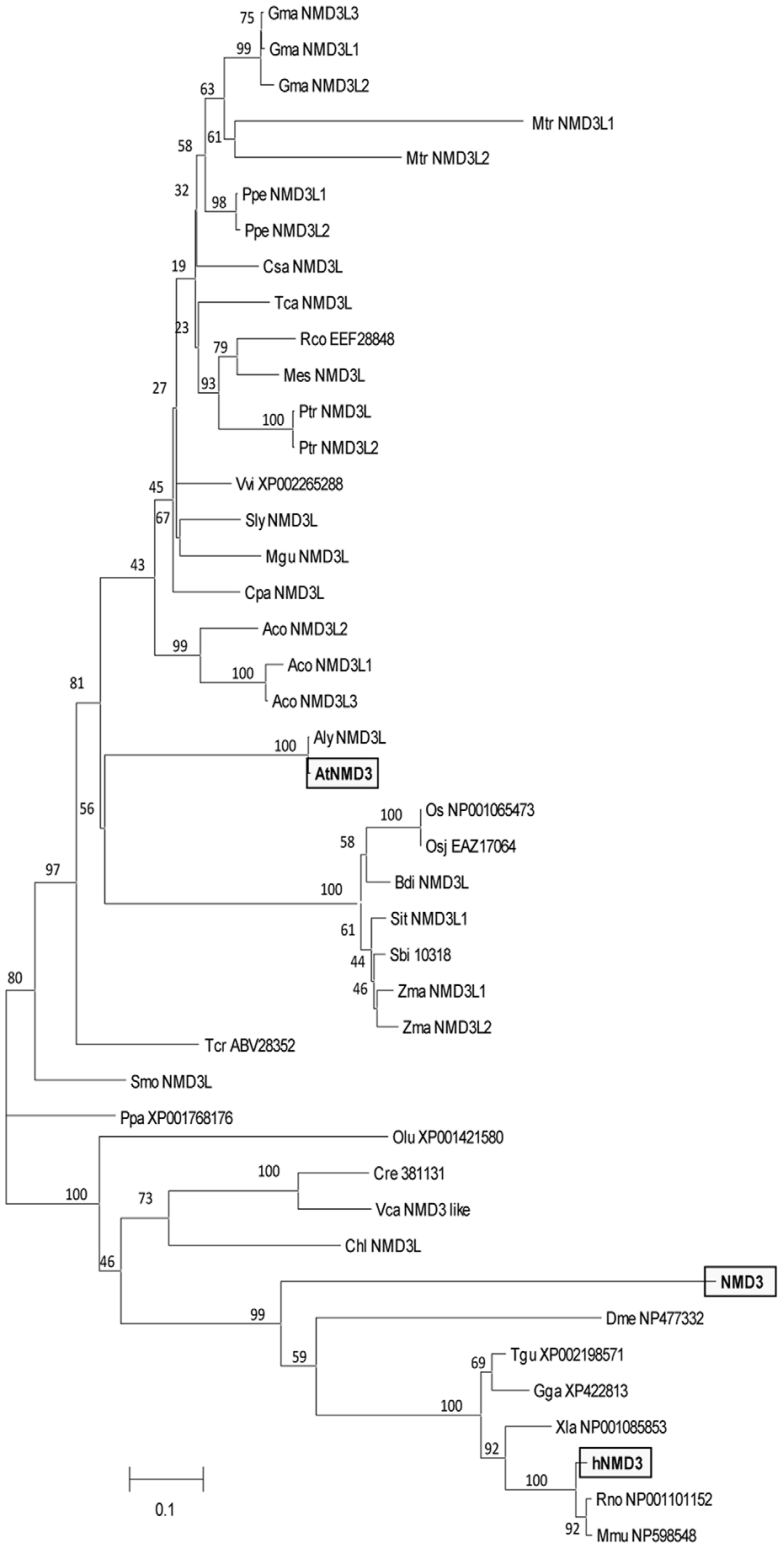
Unrooted phylogenetic analysis of the NMD3 family. Protein sequences of *A. thaliana* NP178476 and *S. cerevisiae* NP012040 were used to search for NMD3 homologs in available genome sequences of organisms (http://www.phytozome.net/, http://blast.ncbi.nlm.nih.gov/). Ptr indicates proteins from poplar (*Populus. trichocarpa*); Vvi (*Vitis vinifera*); Mtr (*Medicago truncatula*); Gma (Glycine max); Zma (*Zea mays*); Osa (*Oryza sativa*); Osj (*Oryza sativa Japonica*); Csa (*Cucumis sativus*); Ppa (*Physcomitrella patens*); Ppe (Prunus persica ); Olu (*Ostreococcus lucimarinus*); Ath (*Arabidopsis thaliana*); Aly (*Arabidopsis lyrata*); Rco (*Ricinus communis*); Tca (*Theobroma cacao*); Sbi (*Sorghum bicolor*); Tcr (*Taiwania cryptomerioides*); Mes (*Manihot esculenta*); Tgu (*Taeniopygia guttata*); Mus (*Mus musculus*); Sly (*Salanum lycopersicum*); Xla (*Xenopus laevis*); Gga (*Gallus gallus*); Mgu (*Mimulus guttatus*); Rno (*Rattus norvegicus* ); Dme (*Drosophila melanogaster*); Sce (*Saccharomyces cerevisiae*); Cre (*Chlamydomonas reinhardtii*); Has (*Homo sapiens*); Cpa (*Carica papaya*); Aco (*Aquilegia coerulea*); Bdi (*Brachypodium distachyon*); Sit (*Setaria italica*); Smo *(Selaginellae moellendorfii*); Vca (*Volvox_carteri*); and Chl (Chlorella).

To systematically analyze the function of AtNMD3, we carried out a phylogenetic analysis using the neighbor-joining method with 36 representative species, including 28 photosynthetic organisms (Figure 1). The result indicated that the NMD3 homolog is widely present in photosynthetic organisms. The sequence comparison showed that within the 516 amino acid AtNMD3, its N terminal sequence (1–150 aa) is highly conserved with its homologs in other species (Figure S1). Further analysis of AtNMD3 showed that this protein contains both leucine-rich nuclear export signals (NES, 453–472 aa and 481–500 aa) and a nuclear localization signal (NLS, 396–428 aa. Figure S3). Together, this sequence information suggested that AtNMD3 may have a function in the nuclear export of the 60S ribosomal subunit similar to that of other NMD3(s) reported in yeast and vertebrate cells.

### AtNMD3 Proteins Shuttle between the Nucleus and Cytoplasm

**Figure 2:**
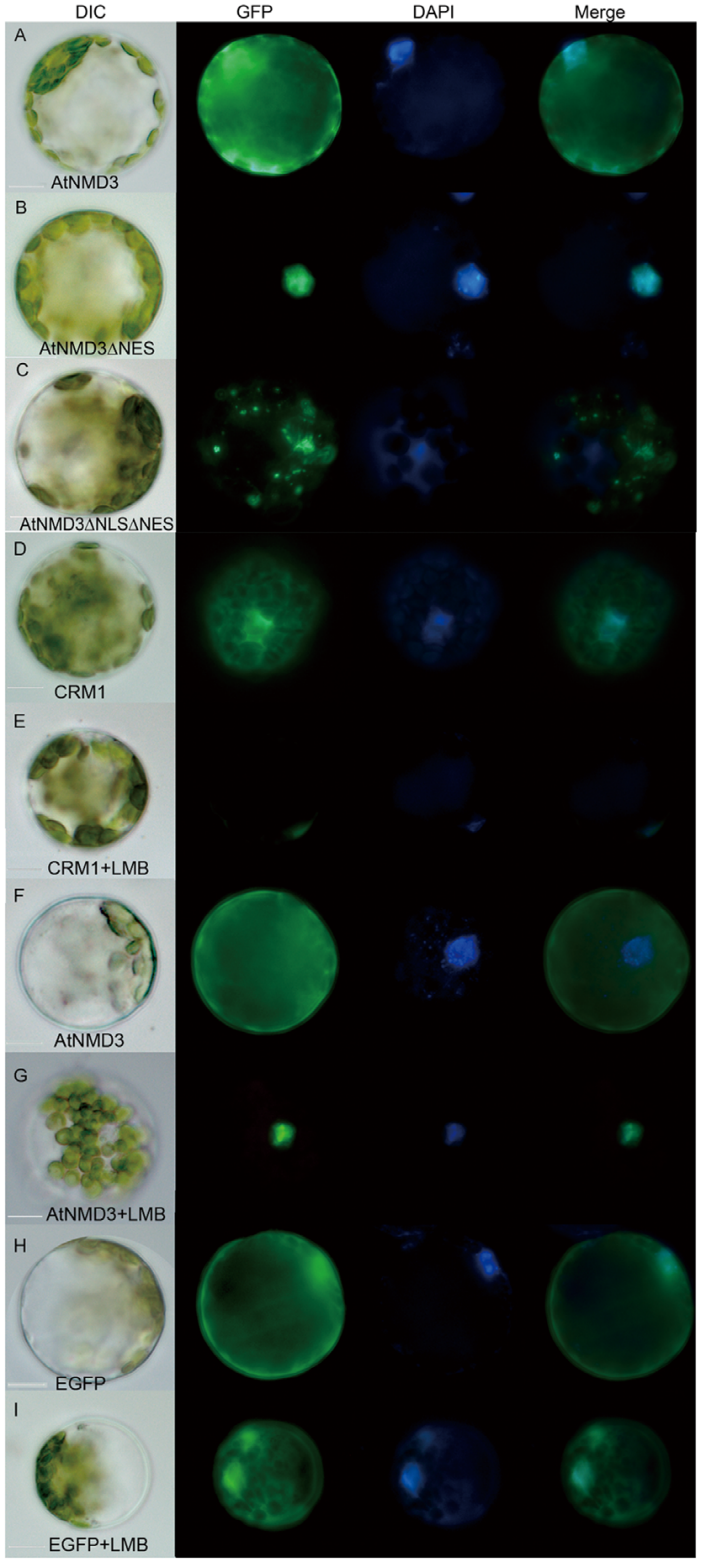
AtNMD3 shuttles between the nucleus and cytoplasm and is inhibited by LMB. **A** to **C** show the localization of EGFP labeled AtNMD3, AtNMD3ΔNES and AtNMD3ΔNLSΔNES in transformed protoplasts, respectively. AtNMD3 was detected in both the nucleus and cytoplasm (**A**), AtNMD3ΔNES was centralized in the nucleus (**B**), and AtNMD3ΔNLSΔNES was distributed in a puntate pattern (**C**). **D** to **G** show the effects of LMB on AtNMD3 localization. As a positive control, EGFP labeled XPO1/CRM1 was detected in both the nucleus and cytoplasm (**D**), but the signals could only be detected in the nucleus after LMB treatment (**E**). LMB changed the nuclear/cytoplasmic distribution of AtNMD3 (**F**), restricting it to the nucleus (**G**). **H** and **I** show that the EGFP distribution was not affected by LMB treatment.

To test whether the AtNMD3 protein functions in the export of 60S ribosomal subunits from the nucleus to the cytoplasm, protoplasts were prepared and transiently transformed with constructs expressing EGFP fusions of AtNMD3 (*35*S*::EGFP-AtNMD3*), AtNMD3 without the C-terminal sequence containing the two predicted NESs (*35*S*::EGFP-AtNMD3ΔNES*) and AtNMD3 lacking both the NES and NLS (*35*S*::EGFP-AtNMD3ΔNLSΔNES*. Figure S4). We found that the full-length AtNMD3 fusion protein could localize to both the nucleus and cytoplasm (Figure 2A), while the AtNMD3 fusion protein without the NESs could only be detected in the nucleus and not in the cytoplasm (Figure 2B). Interestingly, the distribution of the AtNMD3 fusion protein without both the NES and NLS showed a punctuated distribution pattern (Figure 2C), the reason for which needs to be further investigated.

According to the current model, the nuclear export of 60S ribosomal subunits by NMD3 requires formation of a protein complex with XPO1/CRM1 [19]. It has been reported that *Arabidopsis* XPO1/CRM1, similar to those found in yeast and human, is involved in the nuclear export of proteins, and this function is inhibited by the cytotoxin leptomycin (LMB) [20]. If AtNMD3 indeed has an adaptor function for nuclear export of the 60S ribosomal subunit similar to that found in yeast and vertebrate cells, LMB-mediated inhibition of protein export from the nucleus by XPO1/CRM1 should retain the AtNMD3 proteins in the nucleus as well [36], [37]. To test this prediction, we detected the distribution of AtNMD3 upon treatment with LMB. The results showed that the distribution of transiently expressed XPO1/CRM1 (Figure S4) in the cytoplasm of protoplasts was inhibited by LMB (Figure 2D, E). Accordingly, the distribution of AtNMD3 in the cytoplasm was inhibited by LMB as well (Figure 2F, G), while that of EGFP was not affected (Figure 2H, I). These results indicated that AtNMD3 can shuttle between the nucleus and cytoplasm as can XPO1/CRM1.

### AtNMD3 Interacts with 60S Ribosomal Subunits and Affects Their Nuclear/Cytoplasmic Distribution

In yeast, the interaction between NMD3 and the ribosomal protein RPL10 has been reported to be required for release of NMD3 from the ribosome in the cytoplasm [38]–[41]. To clarify how AtNMD3 interacts with the 60S ribosomal subunit, we carried out yeast two-hybrid screening for potential interacting ribosomal proteins (Figure S5). We found AtNMD3 interacted with *Arabidopsis* RPL15, the homolog of yeast RPL28, not with the *Arabidopsis* homolog to RPL10 (Figure S6A). According to the protein structure analysis, we found that yeast RPL28 is localized opposite to the RPL10 in the yeast ribosome (Figure S6B). Perhaps due to this difference, we failed to complement the yeast NMD3 mutant with AtNMD3, even though they have a significantly high protein sequence similarity (Figure S1 and S7).

**Figure 3:**
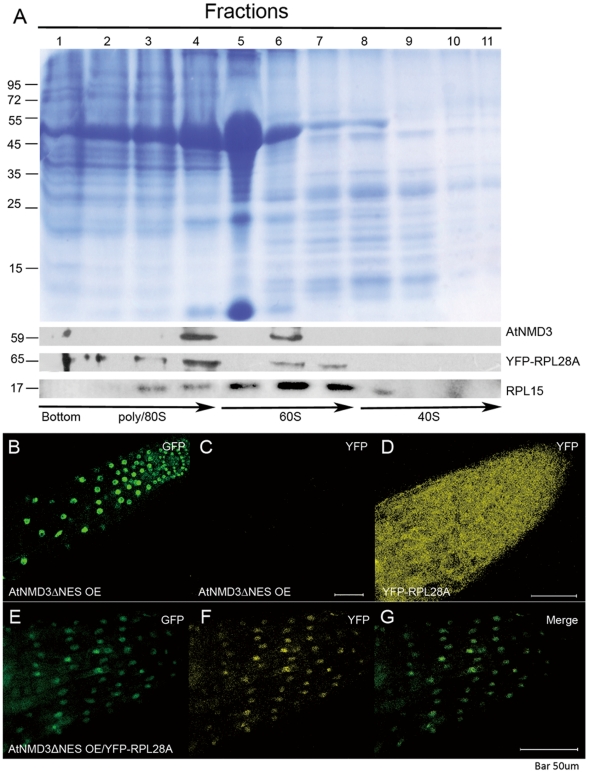
AtNMD3 affects the nuclear export of 60S ribosomal subunits. **A**. Immuno-detection of AtNMD3 proteins co-precipitated with 60S ribosomal subunits. The Commassie stained gel shows the separation of proteins fractionally collected after sucrose density centrifugation. The lower panels show the immune-detection of AtNMD3, YFP-RPL28A and RPL15. While RPL28A and RPL15 could be detected in all fractions containing 60S, the AtNMD3 was co-localized to some fractions containing 60S and 80S, but not 40S. **B** to **G** show the effects of AtNMD3 on RPL28A, a confirmed component of 60S. The EGFP labeled AtNMD3ΔNES was detected in the nuclei of root cells of the stable transgenic lines (Figure S8) at 488/509 nm (for EGFP) (**B**), but not at 515/535 nm (for YFP) (**C**). The YFP labeled RPL28A was detected in both the nuclei and cytoplasm of root cells at 515/535 nm (**D**). In the F1 cross of the two lines, both EGFP (**E**) and YFP (**F**) were centralized in the nuclei, suggesting that the RPL28A together with the 60S subunits were retained in the nuclei due to expression of AtNMD3ΔNES.

If AtNMD3 does in fact interact with ribosomal proteins *in planta*, we believed that it would most likely co-localize with the 60S ribosomal subunits. To verify this prediction, we used inducible RPL28A-YFP transgenic *Arabidopsis* [8] as source material for the collection and separation of YFP tagged ribosomes by sucrose density centrifugation. The fractionated samples were probed with antibodies against GFP (which also recognizes YFP), RPL15 and AtNMD3. Figure 3A shows co-localization of AtNMD3 with 60S as well as 80S ribosome components but not with 40S. This result further suggested that the AtNMD3 interacts with 60S ribosomal subunits.

To determine whether AtNMD3 is required for the nuclear export of 60S ribosomal subunits *in planta*, we crossed the transgenic *Arabidopsis* line overexpressing truncated AtNMD3 without the NES (the AtNMD3ΔNES OE line, Figure S8) with that containing RPL28A-YFP. We found that in the parental AtNMD3ΔNES OE line, the GFP signals could be detected only in the nuclei, and no yellow florescence was observed (Fig 3B, C). Meanwhile, in the RPL28A-YFP lines, the YFP signals could be detected in both the nuclei and cytoplasm (Figure 3D). However, in the F1 plants, the YFP signals could be clearly enriched in the nuclei (Figure 3E, F). This result indicated that AtNMD3 is required for the nuclear export of 60S ribosomal subunits *in planta*.

### Overexpression of AtNMD3ΔNES Results in Defective Secondary Cell Wall Thickening

Although the role of NMD3 in the nuclear export of 60S ribosomal subunits has been well demonstrated in yeast and vertebrate cells, little is known about the effect of aberrant NMD3 function because *NMD3* knockdown is lethal in both systems. In our pilot experiment, we found that in the T1 generation of *AtNMD3* RNAi transgenic plants, there were pleiotropic phenotypes in plant development (Figure S2). In the T2 generation, we were not able to identify individuals with downregulated *AtNMD3* at the RNA level in antibiotic resistant plants (data not shown). We were also not able to find any *AtNMD3* RNA downregulation in the three available homozygous mutants, Cs849934, Salk_146277C (from Salk) and Pst14457 (from RIKEN) (Figure S9). These results implied that on the one hand, AtNMD3 may be indispensable for survival of plants as it is in yeast and vertebrates. On the other hand, a quantitative change in AtNMD3 levels may affect some aspects of plant development. To test the latter possibility, we adopted a dominant negative strategy to further analyze phenotypes of transgenic plants with overexpressed *AtNMD3ΔNES* (AtNMD3ΔNES OE line) that interferes with normal AtNMD3 function (Figures 2, 3; Figure S8).

**Figure 4:**
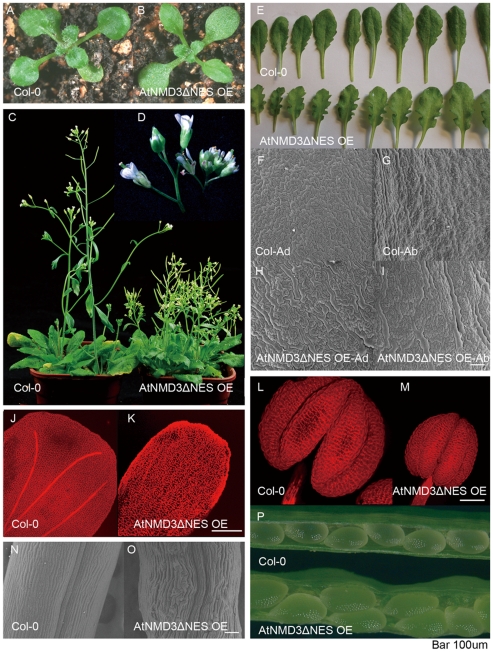
Dominant negative interference of AtNMD3 function causes pleiotropic morphogenetic defects. **A**, **B**. Seventeen-day-old seedlings of wild-type Colombia (Col-0) (**A**) and AtNMD3ΔNES OE line (**B**) show similarities in the overall shape. **C** shows the dwarf phenotype of the AtNMD3ΔNES OE line (right). **D**. Enlarged image of inflorescence (Col-0 at left). **E** shows the rosette leaves of the AtNMD3ΔNES OE line having an obvious zig-zag margin. **F** to **I** show obviously elongated cells in both adaxial and abaxial leaf sides of the AtNMD3ΔNES OE lines (**H**, **I**), compared to Col-0 (**F**, **G**). **J**, **K** shows the petals of the AtNMD3ΔNES OE lines with less organized cells and no obvious vascular veins (**K**), compared to that of Col-0 (**J**). **L**, **M** shows reduced stamen of the AtNMD3ΔNES OE lines (**M**) compared to Col-0 (**L**). **N** and **O** show curved carpel cells of the AtNMD3ΔNES OE lines (**O**), compared to Col-0 (**N**). **P** shows enlarged seeds of the AtNMD3ΔNES OE lines. Bar = 100 µm.

Indeed, pleiotropic phenotypes were observed in the dominant negative transgenic *Arabidopsis* AtNMD3ΔNES OE line (Figure 4). Although the seedlings appeared roughly normal (Figure 4A, B), the AtNMD3ΔNES OE line showed dramatic differences in plant height and particularly in the length of internodes of the inflorescences after flowering compared with wild type plants (Figure 4C, D). Further observation revealed that all above-ground lateral organs showed some abnormalities in the AtNMD3ΔNES OE line, such as obvious curly shape with zig-zag leaf margin of rosette leaves (Figure 4E), extra long-shaped cells on both the adaxial and abaxial sides of the leaf epidermis (Figure 4F-I), lack of obvious vascular veins in petals (Figure 4J, K), reduced stamen size (Figure 4 L, M), curved cells in carpel (Figure 4N, O) and obviously larger seeds (Figure 4P).

**Figure 5:**
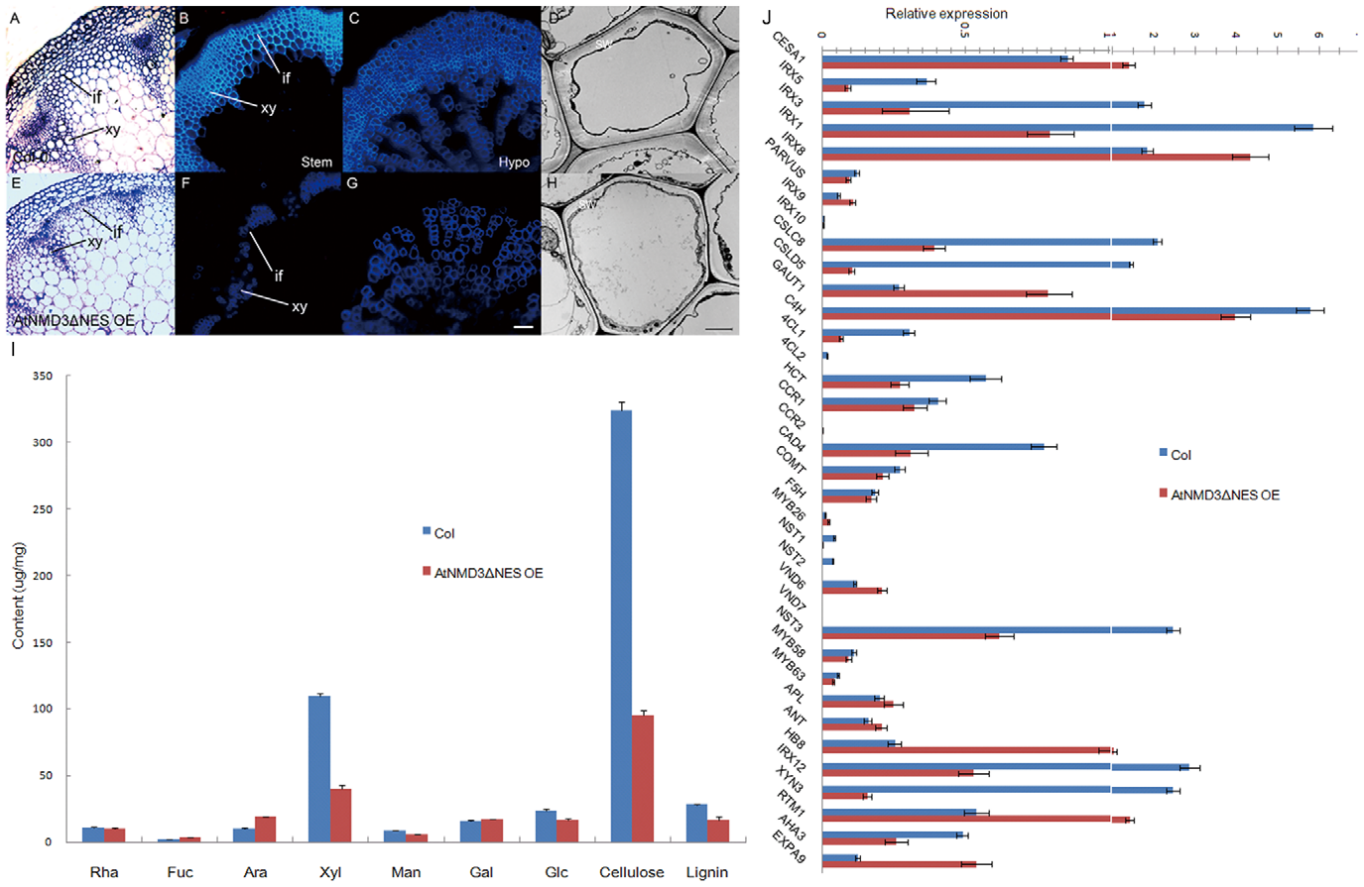
Secondary cell wall thickening is severely defective in the AtNMD3-ΔNES OE line. **A** to **H** show the defect of secondary cell wall thickening in the AtNMD3ΔNES OE line. **A** to **D** show normal morphology of the Col-0 stem after semi-thin plastic sectioning (**A**) and free-hand sectioning for UV observation of the secondary cell walls (**B**), of Col-0 hypocotyl after free-hand sectioning (**C**), and of Col-0 stem after ultra-thin sectioning for TEM observation (**D**). **E** to **H** show abnormal morphology of counterpart samples collected from the AtNMD3ΔNES OE lines, indicating the defect of the secondary cell wall thickening. **I** shows the quantification of the cell wall components, indicating dramatic reductions of xylan (Xyl), cellulose and lignin. **J** shows the expression levels of cell wall related genes. (if: interfascicular fiber; xy: xylem; SW: secondary cell wall. Bar for A, B, C, E, F, G = 50 µm; D, H = 2 µm).

To understand how expression of the dominant negative *AtNMD3* in the AtNMD3ΔNES OE line resulted in such pleiotropic phenotypes, we carefully analyzed the phenotypes. Although the dwarfism (Figure 4C, D) could be caused by numerous defects, lack of veins in the petal (Figure 4J, K) suggested a possible defect in cell wall thickening. Considering the cell wall is a physical boundary for proper cell enlargement in morphogenesis, the defect in cell wall thickening may result in abnormal cell enlargement (Figure 4F–I) and organ enlargement (Figure 4E, P) as observed in the AtNMD3ΔNES OE line. To test this possibility, we observed the cell wall thickening in the AtNMD3ΔNES OE line. In Figure 5A and E, the xylem tissue in the stem vascular bundles and interfascicular fiber cells of the transgenic plants appeared under-differentiated comparing to that in the wild-type. Consistent with this observation, UV illumination revealed that the secondary cell wall thickening was obviously defective not only in the stem (Figure 5B, F), but also in the hypocotyls (Figure 5C, G). Transmission electron microscopy (TEM) examination further confirmed that the secondary cell wall was significantly defective in the interfascicular fiber cells of the stem (Figure 5D, H).

To verify the morphological changes during secondary cell wall thickening, we analyzed the cell wall composition. As shown in Figure 5I, the cellulose content of the AtNMD3ΔNES OE line was dramatically reduced by ∼70%, and that of xylose, which represents the major hemicellulose component xylan, was also decreased by more than 50%. Combined with the reduced lignin level, all these observations suggest that the transgenic lines have abnormal secondary cell wall compositions, consistent with the defects described above.

To determine the molecular basis of the changes in the cell wall composition in the AtNMD3ΔNES OE line, we examined the expressions of the major known genes responsible for cell wall synthesis at the RNA level by quantitative RT-PCR. We found that the transcriptional factor *SND1/NST3*, which acts as a master regulator for secondary cell wall formation, was down-regulated in the AtNMD3ΔNES OE line (Figure 5J). Obviously reduced expressions of genes responsible for cellulose synthesis of secondary cell wall (*CESA4/IRX5*, *CESA7/IRX3*, and *CESA8/IRX1*) and lignin synthesis (*4CL*s, *C4H*, *CAD4*, *COMAT*) (Figure 5J) were also observed. These changes in gene expression were consistent with the alterations in cell wall composition. However, the examined genes for xylan synthesis were not clearly altered. Moreover, the genes responsible for cellulose synthesis of the primary cell wall (*CESA1*), for pectin synthesis (*GUAT1*), as well as for phloem and cambium formation (*APL*, *RTM1*, *HB8* and *ExpA9*) showed increased levels of expression. These findings suggest a compensating response to the inhibited secondary cell wall thickening for maintaining the basic growth of plants.

Taken together, the data above revealed that the dominant negative interference of nuclear export of 60S ribosomal subunit resulted in the defect of the secondary cell wall thickening, which explains the pleiotropic phenotypes observed in the AtNMD3ΔNES OE line.

### The Defective Nuclear Export of 60S Ribosomal Subunit Affects the Formation of RER in the Transgenic Plants and May be Responsible for the Deficient Secondary Cell Wall Thickening

A remaining question is how the defect in nuclear export of the 60S ribosomal subunit might selectively affect secondary cell wall thickening. One possible explanation is that the 60S subunits retained in the nucleus could decrease protein synthesis by ribosomes, thereby impacting the requirements of the secondary cell wall formation. An alternative explanation, which is not mutually exclusive of the previous one, would be that the 60S subunits retained in the nucleus could affect the proper distribution of ribosomes in the cytoplasm or the aggregation of polysomes on the ER (as observed during cotton fiber development [34]), which is required for secondary cell wall thickening.

**Figure 6:**
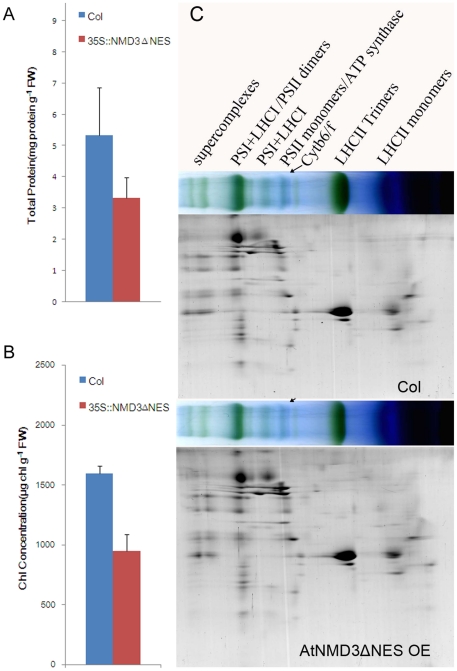
Protein content of the photosynthesis machinery is reduced in the AtNMD3ΔNES OE line. **A**. Total leaf protein content, showing a reduction of protein content in the AtNMD3ΔNES OE line compared to that in the wild-type (Col). The difference between the AtNMD3ΔNES OE line and the wild-type was statistically significant at the level of p<0.05 (n = 5). **B**. Leaf chlorophyll content, showing a proportional reduction of leaf chlorophyll in the AtNMD3ΔNES OE line compared to the wild-type. The difference between the AtNMD3ΔNES OE line and the wild-type was statistically significant at the level of p<0.05 (n = 5). **C**. The complexes of thylakoid membranes analyzed by Blue Native electrophoresis and subsequent SDS-PAGE, showing no obvious difference in the photosynthetic apparatus between the AtNMD3ΔNES OE line and the wild-type.

To test the first possibility described above, we first detected the protein content in the wild-type and the AtNMD3ΔNES OE line. Figure 6 A shows that the total leaf protein content was indeed significantly reduced in the AtNMD3ΔNES OE lines compared to the wild-type. Figure 6B shows that there was a significant decrease in the leaf chlorophyll content in the AtNMD3ΔNES OE line compared to the wild-type. However, the reduction in both total leaf protein and chlorophyll contents did not appear to affect the protein compositions of the thylakoid membranes significantly when analyzed by BN-PAGE on the basis of thylakoid protein content (Figure 6C). Furthermore, no significant differences were observed in the compositions of the main chlorophyll-protein complexes, such as photosystems I and II, between AtNMD3ΔNES OE line and the wild-type plants. If this finding were to be applicable universally in other biological processes in the AtNMD3ΔNES OE line, it would be difficult to explain why only the secondary cell wall thickening process was selectively affected, unless to hypothesize that the secondary cell wall thickening required a dramatic increase in protein synthesis that could not be met in the AtNMD3ΔNES OE line.

**Figure 7:**
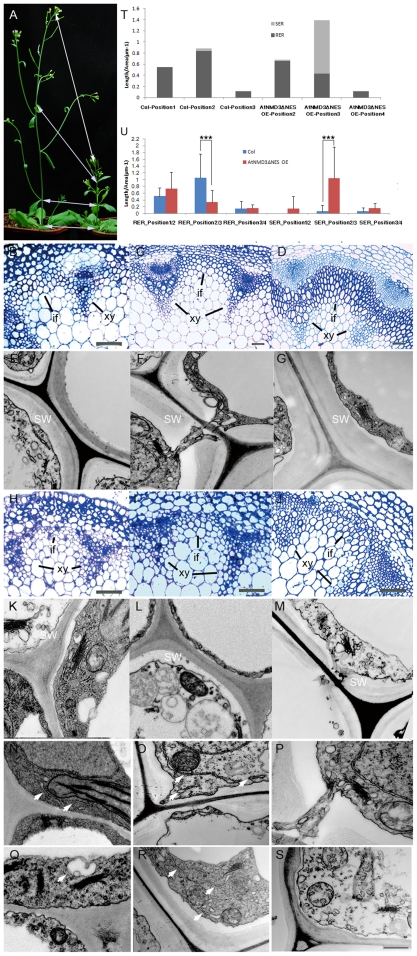
RER formation is reduced in the AtNMD3ΔNES OE line. **A** shows the four morphologically comparable positions (1–4, see text) where the stem samples were collected for observation of the secondary cell wall thickening. **B** to **M** show the morphological features of xylem and interfascicular fibers at positions 2–4 of the Col-0 (**B–D**) and AtNMD3ΔNES OE lines (**H–J**), respectively, and the secondary cell walls at position 2–4 of the Col-0 (**E–G**) and AtNMD3ΔNES OE lines (**K–M**), respectively. **N** to **S** show dynamic changes of ER (arrow heads) in the interfascicular fiber cells at positions 2–4 of the Col-0 (**N–P**) and AtNMD3ΔNES OE lines (**Q–S**), respectively (detailed information see Figure S10 and Table S2). **T**. Relative length of RER and SER in the interfascicular fiber cells at comparative positions between Col and AtNMD3ΔNES OE lines. **U**. Comparison of RER and SER in the comparable cells of Col and AtNMD3ΔNES OE lines. (if: interfascicular fiber; xy: xylem; SW: secondary cell wall. Bars for B, C, D, H, I, J = 50 µm; for E, F, G, K-S = 500 nm. P<0.01; Sample numbers for Col position 1: n = 17, Col position 2: n = 24, Col position 3: n = 8, AtNMD3ΔNES OE position 2: n = 19, AtNMD3ΔNES OE position 3: n = 33, AtNMD3ΔNES OE position 4: n = 5).

To test the second possibility, we analyzed whether there was an abnormal distribution of ribosomes during secondary cell wall thickening in the AtNMD3ΔNES OE line. According to Rogers and Campbell [42] and Ye et al [43], the process of secondary cell wall thickening is best observed in the interfascicular fibers. Considering the dwarf phenotype of the AtNMD3ΔNES OE line and its potential differences from the wild-type in cellular differentiation along with the stem elongation, we arbitrarily collected the samples from the plants with 15 internodes at four particular positions to ensure comparability of the stem samples: the first position was the internode below the flower just opened on the sampling day; the second position was the internode below the last cauline leaf; the third position was the internode just below the first cauline leaf; and the fourth position was the internode at the base of inflorescence (Figure 7A). Based on observations of plastic sections and TEM analysis of the samples, we found that the cellular differentiation status in the AtNMD3ΔNES OE line was about one position behind that of the wild-type plants. For example, the cellular differentiation status of position 3 in the AtNMD3ΔNES OE line is roughly comparable with that of position 2 in the wild-type (Figure 7B–M). The following comparisons of ultra-cellular structures were made of the samples from positions 1, 2 and 3 in the wild-type with that from positions 2, 3 and 4 in the AtNMD3-ΔNES OE line, respectively (Figure 7N–S).

In the comparison between the samples from the wild-type and the AtNMD3ΔNES OE line at their respective positions, we further confirmed the secondary cell wall defects in the AtNMD3ΔNES OE line (Figure 7E–G, positions 2–4 of the wild-type and Figure 7K–M, positions 2–4 of the AtNMD3ΔNES OE line). While we did not identify obvious differences in other ultra-cellular structures, we found dynamic changes in the endoplasmic reticulum (ER; Figure 7N–S, indicated by arrows). Using the relative lengths of the ER, both the smooth (SER) or rough endoplasmic reticulum (RER) in the observed cytoplasmic area as a criterion, excluding the areas of plastids and mitochondria, we quantified the dynamic changes in the amount of ER at the interfascicular fiber cells during stem development. Figure 7T (for details see Figure S10) shows that in the wild-type plants, the relative length of the ER was obviously increased in the interfascicular fiber cells at the position 2 internode compared to the position 1 internode, while this was dramatically decreased at the position 3 internode when the secondary cell wall thickening process was completed. The dynamic changes in the amount of ER could also been observed in the AtNMD3ΔNES OE line (Figure 7T). This phenomenon suggests that the amount of ER is correlated with the cellular differentiation along with the stem development (Figure 7A).

It is worth noting, however, that in the wild-type plants, the ER consisted almost entirely of RER while in the AtNMD3ΔNES OE line the ER at position 3, which is developmentally equivalent to the position 2 in the wild-type plants, consisted mainly of SER. We carried out further statistical analysis of the distribution of RER and SER (Table S2). Figure 7U shows that there were no significant differences in terms of the relative length of RER and SER in the comparable cells at the wild-type position 1 vs. AtNMD3ΔNES OE position 2 and the wild-type position 3 vs. AtNMD3ΔNES OE position 4. In contrast, the relative length of the RER was significantly lower in the comparable cells at AtNMD3ΔNES OE position 3 than at the wild-type position 2, and that of the SER was significantly higher in the comparable cells at AtNMD3ΔNES OE position 3 than at the wild-type position 2. These observations suggest that during stem development, the secondary cell wall thickening may require the rapid accumulation of ER and ribosomes to form the RER in order to ensure the rapid build-up of the machinery required for the mass synthesis of cell wall components. Without the proper nuclear export of 60S ribosomal subunits, as in the AtNMD3ΔNES OE line, there would be no appropriate RER formation although the accumulation of ER would still occur. As a result, there would be no proper secondary cell wall thickening.

## Discussion

NMD3 proteins have been demonstrated to be key components in the nuclear export of 60S ribosomal subunits in yeast and vertebrates [36], [37]. Here we demonstrated that the *Arabidopsis* AtNMD3 protein has a similar function. This finding extends the evolutionary conservation of the NMD3 protein from yeast and animals to plants, not only in sequence, but also in function. However, the detailed mechanism of interaction between NMD3 and the 60S ribosomal subunits may differ in yeast and *Arabidopsis*, since AtNMD3 could not be used to complement the yeast NMD3 mutant.

Currently, no information is available on the outcome of the abnormal nuclear export of 60S ribosomal subunit caused by the defect of NMD3 in multicellular organism development [37]. In this study, by using a dominant negative form of AtNMD3 to interfere with its normal function, we found a severe defect in the secondary cell wall thickening in the AtNMD3ΔNES OE transgenic line. This finding revealed at least one outcome of defective NMD3 function in the development of a multicellular organism.

Considering that the ribosome is the site of protein synthesis, the reduced nuclear export of 60S ribosomal subunit should decrease the capacity of protein synthesis. Our analysis revealed that consistent with the observed nuclear trapping of the 60S ribosomal subunit in the AtNMD3ΔNES OE line (Figure 3), the protein content of the photosynthesis machinery was indeed also reduced, while the protein composition remained unchanged (Figure 6). As to why only the secondary cell wall thickening was selectively affected but other biological processes such as photosynthesis could still support life processes in the AtNMD3ΔNES OE line, one simple explanation is that the secondary cell wall thickening is a mass production of quite diversified metabolites in a short period time, which requires a dramatic increase in the quantity of various enzymes. The capacity of the lower amounts of exported 60S ribosomal subunits therefore could not meet the dramatically increased demand of protein synthesis during the secondary cell wall thickening in the AtNMD3ΔNES OE line.

However, the above simple explanation does not explain why other active processes, such as cell dividing in the shoot apical meristem, which also requires rapid protein synthesis, were not severely affected as no obvious abnormality was observed in morphogenesis of the AtNMD3ΔNES OE line. Therefore, we propose a refined explanation that not only amount of 60S ribosomal subunits, but formation of the RER may be a key for the selective effect on secondary cell wall thickening found in the AtNMD3ΔNES OE line. We found that during stem development when the secondary cell wall was thickening, there was a dramatic increase of ER, mainly RER (Figure 7T). Such a structure is rapidly dismissed after the completion of the secondary cell wall formation. In the AtNMD3ΔNES OE line, we found similar increases and decreases of ER, but not of the RER (Figure 7T, U). This suggested that the formation of the RER, in addition to the quantity of ribosome *per se*, plays a key role in the proper formation of the secondary cell wall. Without a proper amount of RER in the AtNMD3ΔNES OE line, the secondary cell wall thickening would be selectively and severely affected. This explanation is consistent with the correlation between polysome aggregation and secondary cell wall thickening observed during cotton fiber development [35].

However, the “refined explanation” would raise another question: how does the nuclear export of 60S ribosomal subunit affect the expressions of the responsible genes? RER formation and the protein synthesis both occur in the cytoplasm, while gene expression occurs in the nucleus. It is obvious that to ensure a proper secondary cell wall thickening, there must be a mechanism to coordinate the synthesis of enzymes in the cytoplasm and the related mRNA transcription in the nucleus. Transcriptional factors that coordinate the expression of related enzyme genes in the nucleus have been identified previously [25]. However, a question still remains as to how these transcription factors properly coordinate the various cellular components to meet the demand for protein synthesis occurring in the cytoplasm. Our observation of the dynamic change in the RER suggested a new possibility that the nuclear export of 60S ribosomal subunit may be involved in the coordination of the related events simultaneously occurring in the nucleus and cytoplasm during secondary cell wall thickening. It can be imagined that while the capacity for synthesis of enzymes responsible for secondary cell wall thickening is insufficient, the level of the corresponding mRNAs should be in surplus. This surplus may signal back to the nucleus by some unknown mechanism to downregulate the transcription of the related genes. This reasoning is consistent with the downregulation of both transcription factors (*NST1*, *NST2*, *SND1/NST3*, *Myb58* and *Myb63*) and enzyme genes (*CESA4/IRX5*, *CESA7/IRX3*, *CESA8/IRX1*, *CSLD5*, *C4H*, *4CL1*, *HCT*, *CCR1*, *CAD4*, and *COMT*) in our experiments (Figure 5J). By contrast, an insufficiency of ribosomes in the cytoplasm may signal back to increase the transcription of ribosomal proteins. We indeed found that all of the detected ribosomal protein encoding genes were upregulated in the AtNMD3ΔNES OE line (Figure S11). Although the signaling mechanism remains elusive, the nuclear export of the 60S ribosomal subunit and RER formation may be a part of this coordinated process.

In our analysis of related gene expression, we found upregulations of *CESA1*, *IRX8*, *GAUT1*, *HB8*, *RTM1* and *EXPA9* to various extends. Considering the functions of these genes in cellulose synthesis of the primary cell wall (*CESA1*), pectin synthesis (*GAUT1*), phloem formation (*HB8* and *RTM1*) and cambium activity (*EXPA9*), we speculated that the upregulation of these genes may compensate for the defect of the secondary cell wall thickening. These compensating gene expressions may enhance the mechanical strength of the cell wall by increasing synthesis of various components and/or differentiation of phloem and cambium. The increased expressions of these genes also suggest that they may not be regulated by the above hypothesized coordinating mechanism if it does exist. Furthermore, these increased gene expressions indicated that the coordination of the protein synthesis and the gene transcription during the secondary cell wall formation must be the result of a complex multi-faceted process, which would need to be further clarified in future studies.

## Materials and Methods

### Growth of Plants

*A. thaliana* was grown at 22°C on MS agar plates or in soil with 12 h light/12 h dark cycles in a Percival growth chamber.

### Seeds, Stains and Antibodies

The inducible RPL28A-YFP transgenic line was kindly provided by Hai Huang (Institute of Plant Physiology and Ecology, CAS, Shanghai China). Seeds of Cs849934 and Salk_146277C were obtained from the Arabidopsis Biological Resource Center (ABRC, USA), and Pst14457 was ordered from the RIKEN Bio Resource Center (Japan). The AtNMD3 antibody was raised against the peptide FEEEDDDDEDDMAAE and RPL15 against VLGKGHLPENKPFVV by the Tianjin Saie Biology Technology Company (Tianjin, China. Figure S12). The GFP antibody was purchased from Beyotime (AG279. Shanghai, China).

### Sequence Analysis Tools

Protein sequences of *A. thaliana* NP178476 and *Saccharomyces cerevisiae* NP012040 were used to search for NMD3 homologs in available genome sequences of various organisms (http://www.phytozome.net/, http://blast.ncbi.nlm.nih.gov/). Multiple sequence alignments were performed using ClustalX (Plate-Forme de Bio-Informatique, Illkirch Cedex, France). We then used Gblocks (http://molevol.cmima.csic.es/castresana/Gblocks_server.html) to eliminate poorly aligned positions and divergent regions of the alignment. The parameters were: minimum number of sequences for a conserved position, 23; minimum number of sequences for a flanking position, 37; maximum number of contiguous non-conserved positions, 5; minimum length of a block, 5; allowed gap positions, with half. The data from 394 aa was then used to perform neighbor-joining analyses with the complete deletion option, using the Jones-Taylor-Thornton (JTT) model, gamma distribution (G) of 1, and 1000 bootstrap replicates test in MEGA version 5 (http://www.megasoftware.net. [44]).

### Plasmid Construction and Transformation

Plasmids containing EGFP-AtNMD3, EGFP-AtNMD3ΔNES, and EGFP-AtNMD3ΔNLSΔNES were constructed by ligating the PCR-amplified cDNA (primers are listed in Table S1) at the *Bam*HI and *Xba*I sites; EGFP-CRM1 and EGFP were cloned at the *Nco*I and *Bam*HI sites between the CaMV 35S promoter and the nopaline synthase terminator in pRTL2 (kindly provided by Li Yi, College of Life Sciences, PKU). Plasmids containing EGFP - AtNMD3 and EGFP-AtNMD3ΔNES for transgenic plant analysis were constructed by inserting the PCR-amplified cDNAs with the 35S promoter into the pCAMBIA2300 vector at *Pst*I sites. All plasmid constructions were confirmed by sequencing.

Transformation of *Arabidopsis* was carried out according to the procedure described by Wang et al [45]. The third generation of transgenic plants was used for examination of phenotypes. Transient expression of exogenous genes in protoplast was carried out according to Yoo et al [46].

To observe the effect of AtNMD3 on the ribosome distribution *in planta*, the AtNMD3ΔNES OE line was crossed with RPL28A-YFP lines. F1 plants (14-days-old, after pre-treatment with 4 µM estradiol for 48 h) were examined for fluorescence signals (excitation = 488 nm and emission = 509 nm for EGFP, excitation = 514 nm and emission = 535 nm for YFP) using confocal laser-scanning microscopy (Leica Microsystems. Germany).

### Preparation of Protoplasts and LMB Treatments

Protoplasts were prepared for observation of subcellular localization of targeting proteins, either directly from the stably transformed *Arabidopsis* lines containing EGFP-AtNMD3 and EGFP-AtNMD3ΔNES, or from transient expression of EGFP-AtNMD3ΔNLSΔNES, EGFP-CRM1 and EGFP. In both cases, the 2nd or 3rd pair of rosette leaves from 2- or 3-week-old seedlings was used to prepare protoplasts according to Yoo et al. [46]. Protoplasts were incubated to express EGFP fusion proteins overnight (16–36 h), followed by treatment with the inhibitor leptomycin B (LMB, 0.1 mg/ml) for 2 h, and then DAPI (1 µg/ml) was added for 30 min. Fluorescence signals (excitation = 488 nm and emission = 509 nm for EGFP, UV for DAPI) in the transformed protoplasts were examined using a spectral microscope.

### Morphological Analysis

Paraffin sectioning was carried out according to Bai et al [47]. UV fluorescence microscopy for observing secondary cell wall was performed according to Yang et al [48]. For TEM, the stem samples were fixed in 3% glutaraldehyde in cacodylate buffer (pH 7.4) for 4 h at 4°C, and then overnight in 1% osmium tetroxide at 4°C. The fixed samples were dehydrated through an alcohol series and embedded in Spurr’s resin. Ultrathin sections were collected in copper grids with a single slot, stained in 1% uranyl acetate and lead citrate, and examined under an electron microscope (JEOL, Tokyo, Japan). The electron photomicrographs of the cells were captured with a cooled CCD unit (XR40; Advanced Microscopy Techniques, Danvers, MA, USA) attached to the microscope.

### Yeast Two Hybrid Assay and Yeast Mutant Complementation

For detailed procedures, see Figure S5, S6, and S7.

### Isolation and Detection of *Arabidopsis* Ribosomes

Ribosomes were extracted from the 14-day-old RPL28A-YFP transgenic *Arabidopsis* seedlings (after pre-treatment with 4 µM estradiol for 48 h) as described by Zanetti et al [49] with minor modifications. The extract mix was centrifuged at 14,000×*g* for 5 min to remove cell debris, and 700 µl of the extract were separated in a 5 ml 7–47% (v/v) exponential sucrose density gradient by ultracentrifugation (4°C, SW55Ti, 275,000×*g*, 2 h). Fourteen fractions of approximately 350 µl were obtained by use of a gradient fractionator at a speed of 16 drops/min. A total of 11 fractions (from 2 to 12) were precipitated by addition of 2 volumes of ethanol, stored overnight at −20°C and centrifuged at 14,000×*g* for 30min. The pellets were resuspended in 80 µl SDS loading buffer. Immunoblots for detection of targeted protein were carried out according to Xu et al [50].

### Determination of Cell Wall Composition

The monosaccharide composition was determined by gas chromatography mass spectrometry (GC-MS), as described previously [51]. In brief, 2 mg of destarched AIRs were hydrolyzed in 2 M trifluoroacetic acid (TFA) at 121°C for 90 min. The acid hydrolysates were air dried and reduced with sodium borohydride (10 mg/ml in 1 M ammonium hydroxide). After acetylation by acetic anhydride, the alditol acetate derivates were extracted in ethyl acetate and analyzed using an Agilent 7890 GC system equipped with a 5975C MSD (Agilent, www.chem.agilent.com). For crystalline cellulose analysis, the remains after TFA treatment were hydrolyzed in Updegraff reagent (acetic acid: nitric acid: water, 8∶1: ∶2 v/v) at 100°C for 30 min. The cellulose content was quantified using an anthrone assay [52]. The lignin content was analyzed according to Kirk and Obst [53] and Hoebler et al [54].

### Quantitative Real-time RT-PCR Analysis of Cell Wall Related Genes

Total RNA was isolated with a Qiagen RNA isolation kit (Germany) from the inflorescence of 6-week-old plants when the first flower opened. Real-time RT-PCR analysis was performed using the first-strand cDNA as a template with the QuantiTect SYBR Green PCR kit (Cat.4367659,ABI, UK). Primers used for PCR analysis are listed in Table S1. UBIQUITIN10 (UBQ10) was detected as a reference.

### Quantification of Total Leaf Protein, Leaf Chlorophyll and Analysis of Photosynthesis Machinery Proteins

Total leaf protein extraction and quantification were carried out according to Conlon and Salter [55], using the Bio-Rad DC protein Assay kit (Bio-Rad Hercules, CA, USA).

The native complexes of thylakoid membranes of chloroplast were analyzed by Blue Native PAGE carried out in the Hoefer™ SE 250 (GE Healthcare) vertical electrophoresis unit, following a modified protocol by Cline and Mori [56]. All steps of the sample preparation were carried out at 4°C with temperature controlled by a cooling circulator (MultiTemp III, GE Healthcare). Solutions for electrophoresis were: cathode buffer I [50 mM Tricine, 15 mM Bis-Tris, pH 7.0 (4°C), 0.02% (w/v) Coomassie Blue], cathode buffer II [50 mM Tricine, 15 mM Bis-Tris, pH 7.0 (4°C)] and anode buffer [50 mM Bis-Tris, pH 7.0 (4°C)]. The run was initiated at 50 V (approximately 1 mA) for 30 min and continued at 2 mA (50–250 V) for 3 h. The cathode buffer was changed from I to II at 1/3 of the electrophoresis for a clean background of the Blue Native gels. To further analyze the changes in separate proteins of the thylakoid complexes, a second dimensional SDS-PAGE was performed. Lanes of the Blue Native gel with separated protein complexes were excised and soaked in SDS sample buffer [50 mM Tris, 5% (w/v) SDS, 20% (w/v) glycerol, 10% (v/v) β-mercaptoethanol, and 8 M urea] for 30 min with gentle shaking at room temperature. Subsequently, lanes of the Blue Native gels were layered onto 1-mm thick 16% SDS polyacrylamide gels containing 2 M urea. The SDS-PAGE was carried out in a Bio-Rad II vertical electrophoresis system. After electrophoresis the gels were fixed and stained with Blue Silver Coomassie Blue for visualization [57].

The content of leaf chlorophyll was determined according to Arnon [58].

### Measurement of the RER and SER and Statistical Analysis

For a detailed description of RER and SER measurements, see Table S2. Statistical analysis was performed based on 3-way ANOVA in R package 2.12.0 [59], followed by the least significant difference test. The significance level was set at p<0.05.

## Supporting Information

Figure S1 Sequence conservation of AtNMD3 with other NMD3 proteins.
(DOCX)

Figure S2 RNAi reduces AtNMD3 and causes failure of endothecium development in the T1 generation.
(DOC)

Figure S3 Analysis of NES and NSL in AtNMD3 proteins.
(DOC)

Figure S4 Plasmids construction for analysis of AtNMD3 shuttling between the nucleus and cytoplasm.
(DOC)

Figure S5 Yeast two-hybrid screening of proteins interacting with AtNMD3.
(DOC)

Figure S6 Sequence similarity of AtRPL15 with yeast RPL28A and a computational model of RPL28A localization in the ribosome.
(DOC)

Figure S7 AtNMD3 does not complement the yeast temperature sensitive *nmd3* mutant.
(DOC)

Figure S8 Construction of the transgenic *Arabidopsis* overexpressing truncated AtNMD3 without a NES (the AtNMD3ΔNES OE line).
(DOC)

Figure S9 Examination of AtNMD3 expression in the commercially obtained *atnmd3* mutants.
(DOC)

Figure S10 Detailed quantitative data for dynamic changes of RER/SER during stem development in the wild-type and AtNMD3-ΔNES OE lines.
(DOC)

Figure S11 Examination of selected *RPL* gene expression during stem development in the wild type and AtNMD3-ΔNES OE line.
(DOC)

Figure S12 Immunoblot detection of antibody against AtNMD3 and RPL15.
(DOC)

Table S1 Oligonucleotides used in this study.
(DOC)

Table S2 Statistical analysis of dynamic changes of RER/SER during stem development in the wild-type and AtNMD3-ΔNES OE line.
(XLS)
