# Supplementary material for: Arabidopsis NMD3 Is Required for Nuclear Export of 60S Ribosomal Subunits and Affects Secondary Cell Wall Thickening
Source: PLoS One. 2012 Apr 27;7(4):e35904. doi: 10.1371/journal.pone.0035904 (PMC3338764; doi:10.1371/journal.pone.0035904)
Supplement: Figure S1 — Sequence conservation of AtNMD3 with other NMD3 proteins. (DOCX) [file pone.0035904.s001.docx]

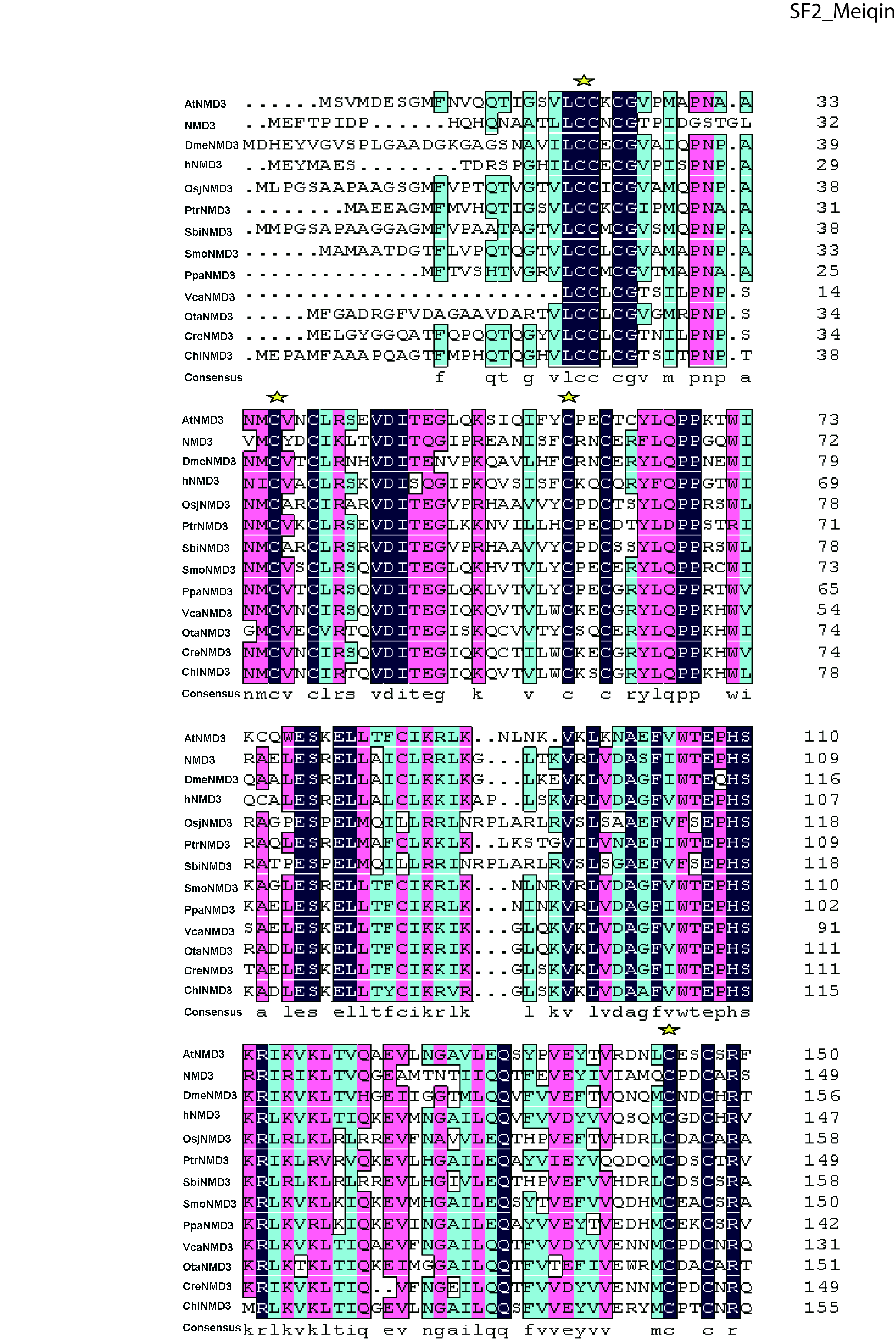


**Figure S1 Sequence conservation of AtNMD3 with other NMD3 proteins**

NMD3 is conserved in photosynthetic organisms. Sequence alignment of the N-terminal domains of Arabidopsis and other 12 species (refer to Figure 1) NMD3 proteins by ClustalW algorithm (Feng-Doolittle and Thompson, <http://www.lynnon.com/pc/alignm.html>) for Optimal Alignment and graphical view output by DNAMAN. Conserved (black) and similar amino acid residues (pink: >75%; greenish blue: >50%) were indicated. Yellow stars show the four conserved Cx2C repeats.
