# Supplementary material for: Arabidopsis NMD3 Is Required for Nuclear Export of 60S Ribosomal Subunits and Affects Secondary Cell Wall Thickening
Source: PLoS One. 2012 Apr 27;7(4):e35904. doi: 10.1371/journal.pone.0035904 (PMC3338764; doi:10.1371/journal.pone.0035904)
Supplement: Figure S2 — RNAi reduces AtNMD3 and causes failure of endothecium development in the T1 generation. (DOC) [file pone.0035904.s002.doc]

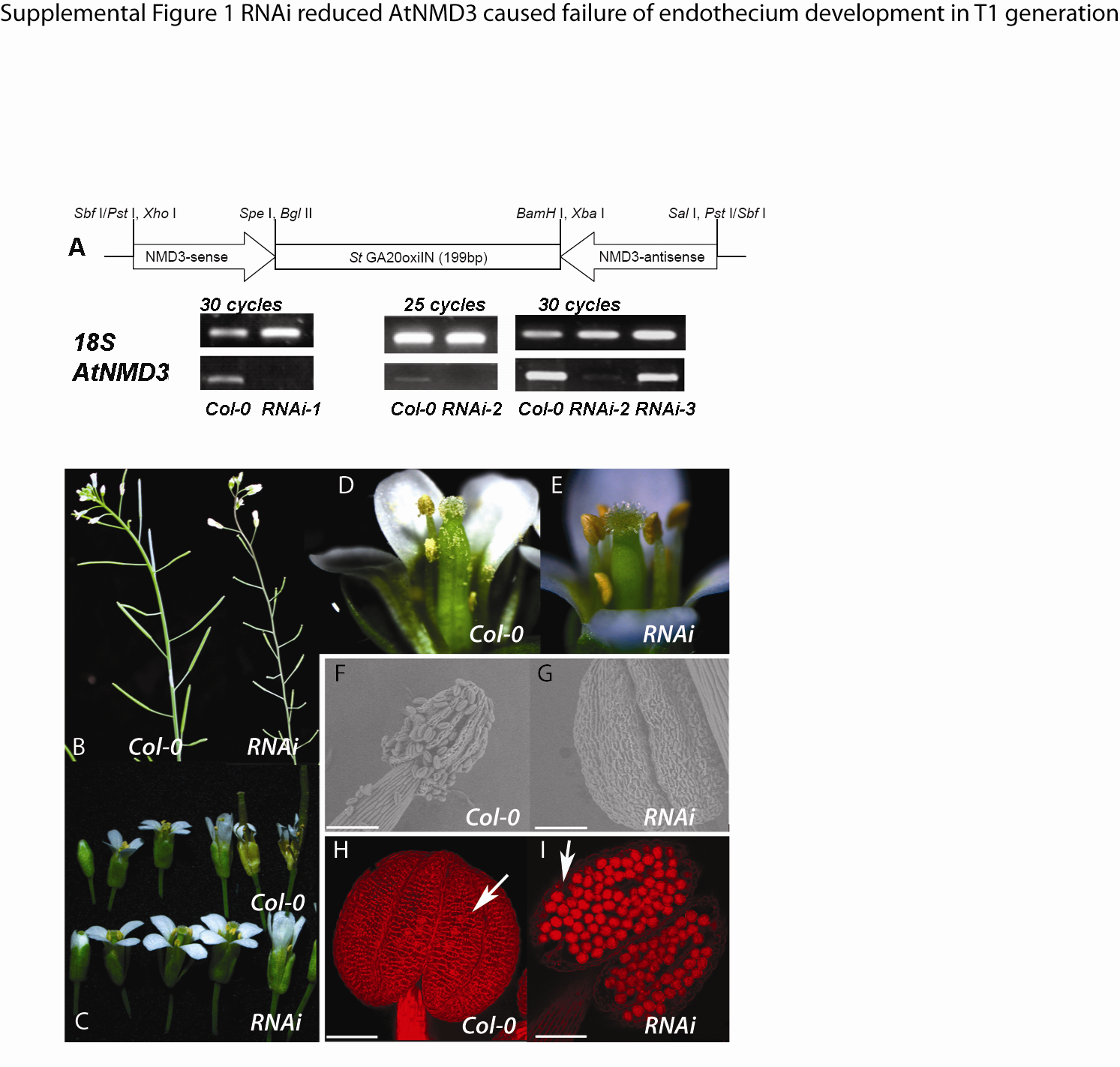


**Figure S2** ***RNAi* reduces *AtNMD3* and causes failure of endothecium development in T1 generation**

1. Scheme of *AtNMD3* RNAi vector construction and RT-PCR Analysis showing *AtNMD3* RNA expression was downregulated in *RNAi-1 and RNAi-2* lines (with sterile phenotype), and not downregulated in *RNAi-3* lines (with no sterile phenotype)
2. *RNAi-1* showed sterile phenotype (Col-0 is wild type Columbia ecotype)
3. *RNAi-1* showed bigger size of flowers than Col-0

**D** and **E.** Pollen were released and pollinated at pistil in Col-0 flowers (**D**), while no pollen release observed in *RNAi-1* line (E)

**F** and **G.** Scanning electron microscopy observation of anther dehiscence in wild-type (**F**) and defect of anther dehiscence in *RNAi-1* lines (**G**) Bar=100μm

**H-I** Secondary cell wall thickening in endothecium is observed in Col-0 (arrow pointed. **H**), not in that of *RNAi-1* line ((arrow pointed, **I**). Anthers were stained with acridine orange/ethidium bromide and visualized by confocal microscopy (excitation, 590 nm). Bar=100μm
