# Supplementary material for: Arabidopsis NMD3 Is Required for Nuclear Export of 60S Ribosomal Subunits and Affects Secondary Cell Wall Thickening
Source: PLoS One. 2012 Apr 27;7(4):e35904. doi: 10.1371/journal.pone.0035904 (PMC3338764; doi:10.1371/journal.pone.0035904)
Supplement: Figure S3 — Analysis of NES and NSL in AtNMD3 proteins. (DOC) [file pone.0035904.s003.doc]

**A**

Seq-Pos-Residue ANN HMM NES Predicted

Sequence-491-I 0.088 0.815 0.518 Yes

Sequence-492-E 0.073 0.815 0.518 Yes

Sequence-493-E 0.072 0.815 0.515 Yes

Sequence-494-L 0.168 0.860 0.575 Yes

Sequence-495-L 0.094 0.917 0.631 Yes

Sequence-496-A 0.151 0.916 0.645 Yes

Sequence-497-D 0.113 0.916 0.650 Yes

Sequence-498-L 0.142 0.919 0.667 Yes

Sequence-499-D 0.097 0.917 0.670 Yes

Sequence-500-L 0.910 0.918 1.238 Yes


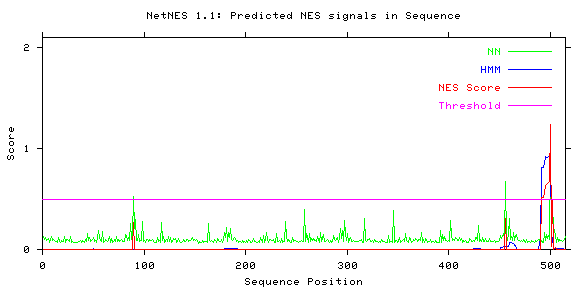


**B**


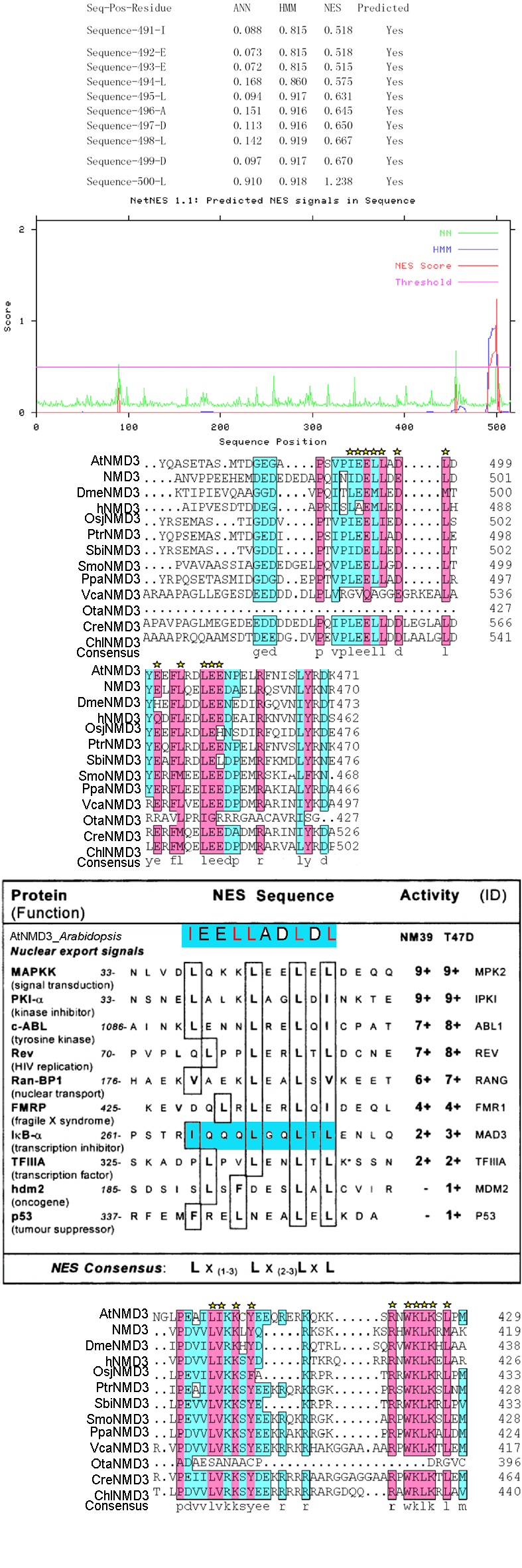


**C**


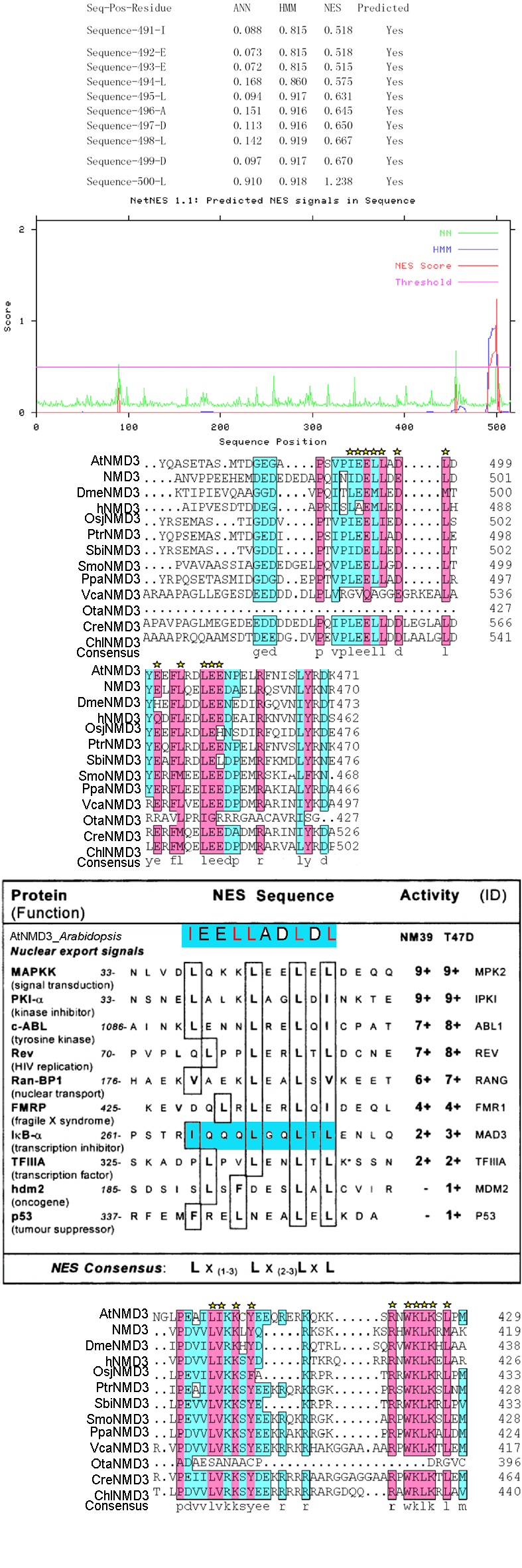


**D**


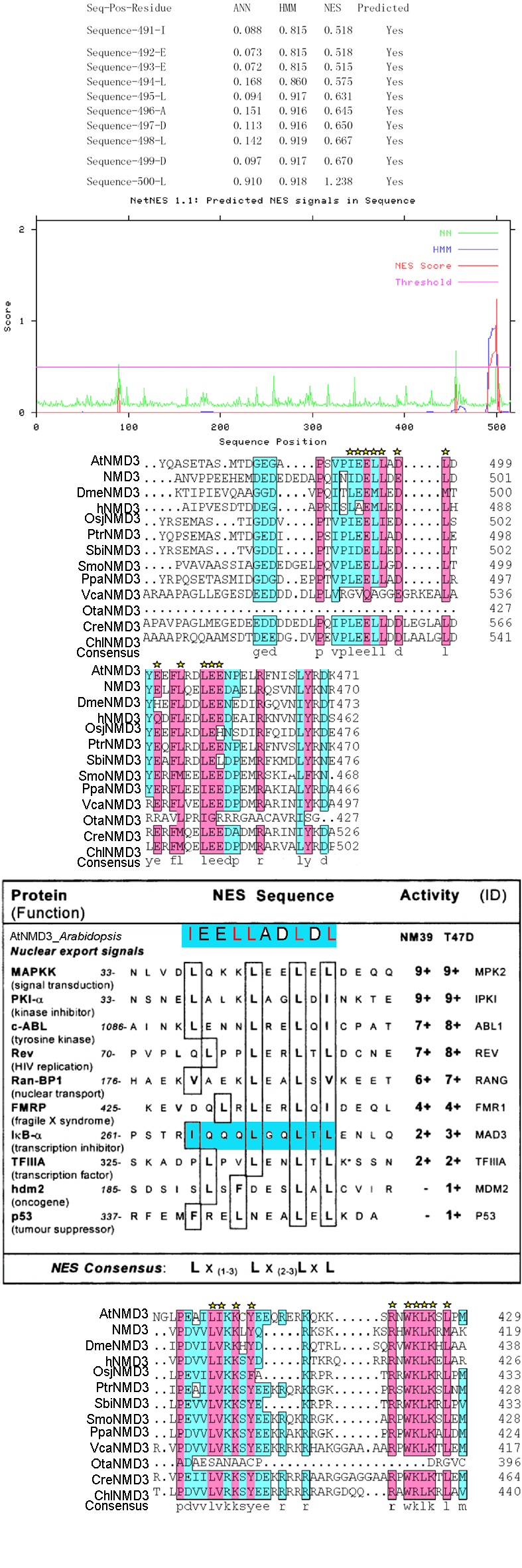


**E**


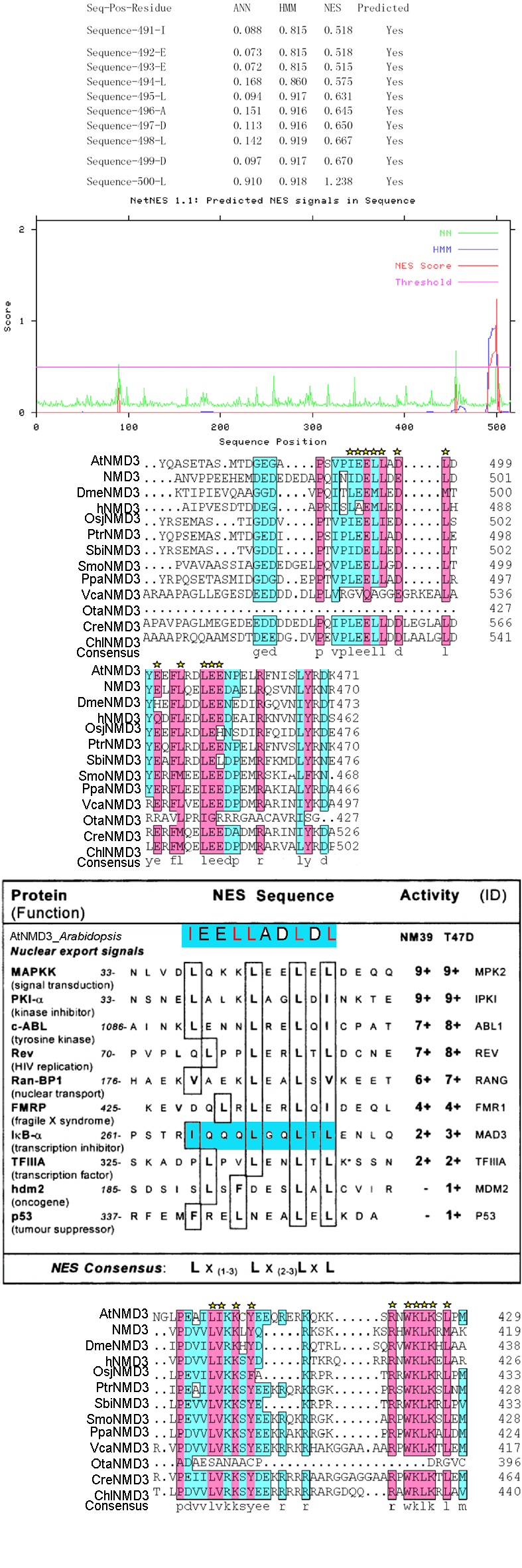


**Figure S3 Analysis of NES and NLS in AtNMD3 protein**

**A.** NES in AtNMD3 protein (491-500AA position) was predicted by NetNES 1.1 Server analysis.

**B.** These NES sequences match criteria of NES detailed analyzed by Henderson and Eleftheriou (2002)

**C - E.** Conserved sequence of NES1 (**C**), NES2 (**D**) and NLS (**E**) of NMD3 in all 13 species (indicated by yellow stars, Sequence alignment were same with Figure S1)

[Henderson BR](http://www.ncbi.nlm.nih.gov/pubmed?term="Henderson BR"%5BAuthor%5D), [Eleftheriou A](http://www.ncbi.nlm.nih.gov/pubmed?term="Eleftheriou A"%5BAuthor%5D). 2000. A comparison of the activity, sequence specificity, and CRM1-dependence of different nuclear export signals. [*Exp Cell Res*.](http://www.ncbi.nlm.nih.gov/pubmed/?term=Henderson+and+Eleftheriou,+2000)**256**:213-24
