# Supplementary material for: Arabidopsis NMD3 Is Required for Nuclear Export of 60S Ribosomal Subunits and Affects Secondary Cell Wall Thickening
Source: PLoS One. 2012 Apr 27;7(4):e35904. doi: 10.1371/journal.pone.0035904 (PMC3338764; doi:10.1371/journal.pone.0035904)
Supplement: Figure S4 — Plasmids construction for analysis of AtNMD3 shuttling between the nucleus and cytoplasm. (DOC) [file pone.0035904.s004.doc]

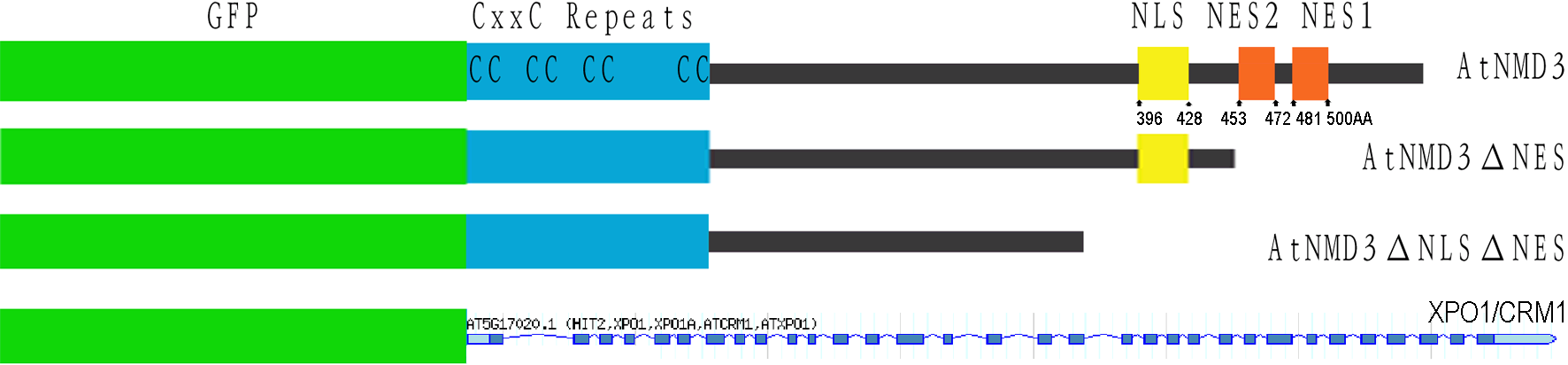


**A**

EGFP

**B**

***35S::EGFP-AtNMD3***

ctgcaggtcAACATGGTGGAGCACGACACTCTCGTCTACTCCAAGAATATCAAAGATACAGTCTCAGAAGACCAGAGGGCTATTGAGACTTTTCAACAAAGGGTAATATCGGGAAACCTCCTCGGATTCCATTGCCCAGCTATCTGTCACTTCATCGAAAGGACAGTAGAAAAGGAAGATGGCTTCTACAAATGCCATCATTGCGATAAAGGAAAGGCTATCGTTCAAGAATGCCTCTACCGACAGTGGTCCCAAAGATGGACCCCCACCCACGAGGAACATCGTGGAAAAAGAAGACGTTCCAACCACGTCTTCAAAGCAAGTGGATTGATGTGATAACATGGTGGAGCACGACACTCTCGTCTACTCCAAGAATATCAAAGATACAGTCTCAGAAGACCAGAGGGCTATTGAGACTTTTCAACAAAGGGTAATATCGGGAAACCTCCTCGGATTCCATTGCCCAGCTATCTGTCACTTCATCGAAAGGACAGTAGAAAAGGAAGATGGCTTCTACAAATGCCATCATTGCGATAAAGGAAAGGCTATCGTTCAAGAATGCCTCTACCGACAGTGGTCCCAAAGATGGACCCCCACCCACGAGGAACATCGTGGAAAAAGAAGACGTTCCAACCACGTCTTCAAAGCAAGTGGATTGATGTGATATCTCCACTGACGTAAGGGATGACGCACAATCCCACTATCCTTCGCAAGACCCTTCCTCTATATAAGGAAGTTCATTTCATTTGGAGAGGACctcgagaattcAACACAACATATACAAAACAAACGAATCTCAAGCAATCAAGCATTCTACTTCTATTGCAGCAATTTAAATCATTTCTTTTAAAGCAAAAGCAATTTTCTGAAAATTTTCACCATTTACGAACGATAGCCATGGCACTCATCTTTGGCACAGTCAACGCTAACATCCTGAAGGAAGTGTTCGGTGGAGCTCGGTACCCGGGGATCCATGGTGAGCAAGGGCGAGGAGCTGTTCACCGGGGTGGTGCCCATCCTGGTCGAGCTGGACGGCGACGTAAACGGCCACAAGTTCAGCGTGTCCGGCGAGGGCGAGGGCGATGCCACCTACGGCAAGCTGACCCTGAAGTTCATCTGCACCACCGGCAAGCTGCCCGTGCCCTGGCCCACCCTCGTGACCACCCTGACCTACGGCGTGCAGTGCTTCAGCCGCTACCCCGACCACATGAAGCAGCACGACTTCTTCAAGTCCGCCATGCCCGAAGGCTACGTCCAGGAGCGCACCATCTTCTTCAAGGACGACGGCAACTACAAGACCCGCGCCGAGGTGAAGTTCGAGGGCGACACCCTGGTGAACCGCATCGAGCTGAAGGGCATCGACTTCAAGGAGGACGGCAACATCCTGGGGCACAAGCTGGAGTACAACTACAACAGCCACAACGTCTATATCATGGCCGACAAGCAGAAGAACGGCATCAAGGTGAACTTCAAGATCCGCCACAACATCGAGGACGGCAGCGTGCAGCTCGCCGACCACTACCAGCAGAACACCCCCATCGGCGACGGCCCCGTGCTGCTGCCCGACAACCACTACCTGAGCACCCAGTCCGCCCTGAGCAAAGACCCCAACGAGAAGCGCGATCACATGGTCCTGCTGGAGTTCGTGACCGCCGCCGGGATCACTCTCGGCATGGACGAGCTGTACAAGATGTCAGTAATGGATGAATCAGGCATGTTTAACGTTCAACAAACCATTGGAAGTGTATTGTGTTGCAAGTGTGGTGTTCCCATGGCACCAAACGCAGCCAACATGTGTGTGAATTGTCTTCGTTCCGAAGTCGATATCACCGAAGGTTTACAGAAGAGTATTCAGATCTTCTATTGCCCTGAATGCACTTGTTACTTACAGCCACCAAAGACTTGGATCAAATGTCAATGGGAATCTAAAGAGCTTTTGACATTTTGTATCAAGAGGTTGAAGAATCTCAATAAGGTTAAGCTGAAGAACGCTGAATTCGTTTGGACTGAGCCTCATTCCAAGAGGATTAAGGTTAAGTTGACTGTTCAAGCTGAGGTTCTTAATGGTGCTGTTCTTGAACAGTCTTATCCTGTTGAGTATACGGTTAGGGATAATCTGTGCGAGTCGTGTTCGAGGTTTCAGGCTAATCCTGATCAGTGGGTTGCTTCTATTCAGCTTAGGCAGCATGTTTCGCATAGGAGGACTTTCTTTTATCTCGAACAGTTGATTCTTAGGCACGATGCTGCTTCACGTGCCATTAGAATCCAGCAGGTGGATCAGGGGATTGATTTCTTCTTTGGGAATAAAAGTCATGCTAATAGCTTTGTGGAGTTTCTGAGGAAAGTTGTCCCCATTGAATACCGTCAGGACCAACAGCTGGTGTCTCATGATGTGAAAAGCAGTTTGTACAACTATAAGTACACTTACTCTGTTAAGATCTGTCCTGTTTGCCGTGAGGATCTTGTTTGCTTGCCTTCTAAAGTTGCTAGCGGCTTGGGGAACCTTGGTCCACTTGTGGTTTGCACAAAAGTCTCTGATAATATCACTCTACTTGATCCGAGAACTCTAAGGTGTGCCTTCTTGGATGCGAGACAGTACTGGAGGTCTGGGTTCCGATCTGCGCTCACCAGTAGACAACTTGTCAAGTACTTTGTGTTTGATGTTGAGCCACCTGTTGGTGAAGCGACTGTTGGTGGGCAAAAGTACGCTCTTTCCTATGTCCAGATTGCCCGTGAATCAGACATTGGTAAGATGTTTTATGTCCAAACTCATCTTGGACACATTCTGAAACCTGGGGATCAAGCTTTGGGTTATGACATCTACGGAGCTAATGTGAACGACAATGAAATGGAGAAATACCGTCTGAGTGTGAAGAATGGGCTTCCTGAAGCAATTCTGATCAAGAAATGTTACGAGGAGCAGAGAGAGAGGAAGCAGAAGAAATCCCGTAACTGGAAACTCAAGTCGCTTCCAATGGAAATGGATGATTCAAGAGGCAGGGTTGATCCAGAGAAGACAGACAAAGAATACGAAGAGTTTTTGAGGGATCTTGAAGAAAACCCTGAGCTAAGGTTCAACATATCTTTGTACAGGGACAAGGATTATCAAGCTTCTGAGACTGCTTCAATGACAGATGGAGAAGGTGCACCATCTGTTCCAATTGAAGAGTTGCTTGCTGACCTTGACCTAAGCTTTGAGGAAGAAGACGATGATGACGAGGACGACATGGCTGCTGAAcTCTAGAgtcCGCAAATCACCAGTCTCTCTCTACAAATCTATCTCTCTCTATTTTCTCCAGAATAATGTGTGAGTAGTTCCCAGATAAGGGAATTAGGGTTCTTATAGGGTTTCGCTCATGTGTTGAGCATATAAGAAACCCTTAGTATGTATTTGTATTTGTAAAATACTTCTATCAATAAAATTTCTAATTCCTAAAACCAAAATCCAGTgacctgcag

***35S::EGFP-AtNMD3ΔNES***

ctgcaggtcAACATGGTGGAGCACGACACTCTCGTCTACTCCAAGAATATCAAAGATACAGTCTCAGAAGACCAGAGGGCTATTGAGACTTTTCAACAAAGGGTAATATCGGGAAACCTCCTCGGATTCCATTGCCCAGCTATCTGTCACTTCATCGAAAGGACAGTAGAAAAGGAAGATGGCTTCTACAAATGCCATCATTGCGATAAAGGAAAGGCTATCGTTCAAGAATGCCTCTACCGACAGTGGTCCCAAAGATGGACCCCCACCCACGAGGAACATCGTGGAAAAAGAAGACGTTCCAACCACGTCTTCAAAGCAAGTGGATTGATGTGATAACATGGTGGAGCACGACACTCTCGTCTACTCCAAGAATATCAAAGATACAGTCTCAGAAGACCAGAGGGCTATTGAGACTTTTCAACAAAGGGTAATATCGGGAAACCTCCTCGGATTCCATTGCCCAGCTATCTGTCACTTCATCGAAAGGACAGTAGAAAAGGAAGATGGCTTCTACAAATGCCATCATTGCGATAAAGGAAAGGCTATCGTTCAAGAATGCCTCTACCGACAGTGGTCCCAAAGATGGACCCCCACCCACGAGGAACATCGTGGAAAAAGAAGACGTTCCAACCACGTCTTCAAAGCAAGTGGATTGATGTGATATCTCCACTGACGTAAGGGATGACGCACAATCCCACTATCCTTCGCAAGACCCTTCCTCTATATAAGGAAGTTCATTTCATTTGGAGAGGACctcgagaattcAACACAACATATACAAAACAAACGAATCTCAAGCAATCAAGCATTCTACTTCTATTGCAGCAATTTAAATCATTTCTTTTAAAGCAAAAGCAATTTTCTGAAAATTTTCACCATTTACGAACGATAGCCATGGCACTCATCTTTGGCACAGTCAACGCTAACATCCTGAAGGAAGTGTTCGGTGGAGCTCGGTACCCGGGGATCCATGGTGAGCAAGGGCGAGGAGCTGTTCACCGGGGTGGTGCCCATCCTGGTCGAGCTGGACGGCGACGTAAACGGCCACAAGTTCAGCGTGTCCGGCGAGGGCGAGGGCGATGCCACCTACGGCAAGCTGACCCTGAAGTTCATCTGCACCACCGGCAAGCTGCCCGTGCCCTGGCCCACCCTCGTGACCACCCTGACCTACGGCGTGCAGTGCTTCAGCCGCTACCCCGACCACATGAAGCAGCACGACTTCTTCAAGTCCGCCATGCCCGAAGGCTACGTCCAGGAGCGCACCATCTTCTTCAAGGACGACGGCAACTACAAGACCCGCGCCGAGGTGAAGTTCGAGGGCGACACCCTGGTGAACCGCATCGAGCTGAAGGGCATCGACTTCAAGGAGGACGGCAACATCCTGGGGCACAAGCTGGAGTACAACTACAACAGCCACAACGTCTATATCATGGCCGACAAGCAGAAGAACGGCATCAAGGTGAACTTCAAGATCCGCCACAACATCGAGGACGGCAGCGTGCAGCTCGCCGACCACTACCAGCAGAACACCCCCATCGGCGACGGCCCCGTGCTGCTGCCCGACAACCACTACCTGAGCACCCAGTCCGCCCTGAGCAAAGACCCCAACGAGAAGCGCGATCACATGGTCCTGCTGGAGTTCGTGACCGCCGCCGGGATCACTCTCGGCATGGACGAGCTGTACAAGATGTCAGTAATGGATGAATCAGGCATGTTTAACGTTCAACAAACCATTGGAAGTGTATTGTGTTGCAAGTGTGGTGTTCCCATGGCACCAAACGCAGCCAACATGTGTGTGAATTGTCTTCGTTCCGAAGTCGATATCACCGAAGGTTTACAGAAGAGTATTCAGATCTTCTATTGCCCTGAATGCACTTGTTACTTACAGCCACCAAAGACTTGGATCAAATGTCAATGGGAATCTAAAGAGCTTTTGACATTTTGTATCAAGAGGTTGAAGAATCTCAATAAGGTTAAGCTGAAGAACGCTGAATTCGTTTGGACTGAGCCTCATTCCAAGAGGATTAAGGTTAAGTTGACTGTTCAAGCTGAGGTTCTTAATGGTGCTGTTCTTGAACAGTCTTATCCTGTTGAGTATACGGTTAGGGATAATCTGTGCGAGTCGTGTTCGAGGTTTCAGGCTAATCCTGATCAGTGGGTTGCTTCTATTCAGCTTAGGCAGCATGTTTCGCATAGGAGGACTTTCTTTTATCTCGAACAGTTGATTCTTAGGCACGATGCTGCTTCACGTGCCATTAGAATCCAGCAGGTGGATCAGGGGATTGATTTCTTCTTTGGGAATAAAAGTCATGCTAATAGCTTTGTGGAGTTTCTGAGGAAAGTTGTCCCCATTGAATACCGTCAGGACCAACAGCTGGTGTCTCATGATGTGAAAAGCAGTTTGTACAACTATAAGTACACTTACTCTGTTAAGATCTGTCCTGTTTGCCGTGAGGATCTTGTTTGCTTGCCTTCTAAAGTTGCTAGCGGCTTGGGGAACCTTGGTCCACTTGTGGTTTGCACAAAAGTCTCTGATAATATCACTCTACTTGATCCGAGAACTCTAAGGTGTGCCTTCTTGGATGCGAGACAGTACTGGAGGTCTGGGTTCCGATCTGCGCTCACCAGTAGACAACTTGTCAAGTACTTTGTGTTTGATGTTGAGCCACCTGTTGGTGAAGCGACTGTTGGTGGGCAAAAGTACGCTCTTTCCTATGTCCAGATTGCCCGTGAATCAGACATTGGTAAGATGTTTTATGTCCAAACTCATCTTGGACACATTCTGAAACCTGGGGATCAAGCTTTGGGTTATGACATCTACGGAGCTAATGTGAACGACAATGAAATGGAGAAATACCGTCTGAGTGTGAAGAATGGGCTTCCTGAAGCAATTCTGATCAAGAAATGTTACGAGGAGCAGAGAGAGAGGAAGCAGAAGAAATCCCGTAACTGGAAACTCAAGTCGCTTCCAATGGAAATGGATGATTCAAGAGGCAGGGTTGATCCAGAGAAGACAGACAAAGAATACGAAGAGTTTcTCTAGAgtcCGCAAATCACCAGTCTCTCTCTACAAATCTATCTCTCTCTATTTTCTCCAGAATAATGTGTGAGTAGTTCCCAGATAAGGGAATTAGGGTTCTTATAGGGTTTCGCTCATGTGTTGAGCATATAAGAAACCCTTAGTATGTATTTGTATTTGTAAAATACTTCTATCAATAAAATTTCTAATTCCTAAAACCAAAATCCAGTgacctgcag

***35S::EGFP-AtNMD3ΔNLSΔNES***

ctgcaggtcAACATGGTGGAGCACGACACTCTCGTCTACTCCAAGAATATCAAAGATACAGTCTCAGAAGACCAGAGGGCTATTGAGACTTTTCAACAAAGGGTAATATCGGGAAACCTCCTCGGATTCCATTGCCCAGCTATCTGTCACTTCATCGAAAGGACAGTAGAAAAGGAAGATGGCTTCTACAAATGCCATCATTGCGATAAAGGAAAGGCTATCGTTCAAGAATGCCTCTACCGACAGTGGTCCCAAAGATGGACCCCCACCCACGAGGAACATCGTGGAAAAAGAAGACGTTCCAACCACGTCTTCAAAGCAAGTGGATTGATGTGATAACATGGTGGAGCACGACACTCTCGTCTACTCCAAGAATATCAAAGATACAGTCTCAGAAGACCAGAGGGCTATTGAGACTTTTCAACAAAGGGTAATATCGGGAAACCTCCTCGGATTCCATTGCCCAGCTATCTGTCACTTCATCGAAAGGACAGTAGAAAAGGAAGATGGCTTCTACAAATGCCATCATTGCGATAAAGGAAAGGCTATCGTTCAAGAATGCCTCTACCGACAGTGGTCCCAAAGATGGACCCCCACCCACGAGGAACATCGTGGAAAAAGAAGACGTTCCAACCACGTCTTCAAAGCAAGTGGATTGATGTGATATCTCCACTGACGTAAGGGATGACGCACAATCCCACTATCCTTCGCAAGACCCTTCCTCTATATAAGGAAGTTCATTTCATTTGGAGAGGACctcgagaattcAACACAACATATACAAAACAAACGAATCTCAAGCAATCAAGCATTCTACTTCTATTGCAGCAATTTAAATCATTTCTTTTAAAGCAAAAGCAATTTTCTGAAAATTTTCACCATTTACGAACGATAGCCATGGCACTCATCTTTGGCACAGTCAACGCTAACATCCTGAAGGAAGTGTTCGGTGGAGCTCGGTACCCGGGGATCCATGGTGAGCAAGGGCGAGGAGCTGTTCACCGGGGTGGTGCCCATCCTGGTCGAGCTGGACGGCGACGTAAACGGCCACAAGTTCAGCGTGTCCGGCGAGGGCGAGGGCGATGCCACCTACGGCAAGCTGACCCTGAAGTTCATCTGCACCACCGGCAAGCTGCCCGTGCCCTGGCCCACCCTCGTGACCACCCTGACCTACGGCGTGCAGTGCTTCAGCCGCTACCCCGACCACATGAAGCAGCACGACTTCTTCAAGTCCGCCATGCCCGAAGGCTACGTCCAGGAGCGCACCATCTTCTTCAAGGACGACGGCAACTACAAGACCCGCGCCGAGGTGAAGTTCGAGGGCGACACCCTGGTGAACCGCATCGAGCTGAAGGGCATCGACTTCAAGGAGGACGGCAACATCCTGGGGCACAAGCTGGAGTACAACTACAACAGCCACAACGTCTATATCATGGCCGACAAGCAGAAGAACGGCATCAAGGTGAACTTCAAGATCCGCCACAACATCGAGGACGGCAGCGTGCAGCTCGCCGACCACTACCAGCAGAACACCCCCATCGGCGACGGCCCCGTGCTGCTGCCCGACAACCACTACCTGAGCACCCAGTCCGCCCTGAGCAAAGACCCCAACGAGAAGCGCGATCACATGGTCCTGCTGGAGTTCGTGACCGCCGCCGGGATCACTCTCGGCATGGACGAGCTGTACAAGATGTCAGTAATGGATGAATCAGGCATGTTTAACGTTCAACAAACCATTGGAAGTGTATTGTGTTGCAAGTGTGGTGTTCCCATGGCACCAAACGCAGCCAACATGTGTGTGAATTGTCTTCGTTCCGAAGTCGATATCACCGAAGGTTTACAGAAGAGTATTCAGATCTTCTATTGCCCTGAATGCACTTGTTACTTACAGCCACCAAAGACTTGGATCAAATGTCAATGGGAATCTAAAGAGCTTTTGACATTTTGTATCAAGAGGTTGAAGAATCTCAATAAGGTTAAGCTGAAGAACGCTGAATTCGTTTGGACTGAGCCTCATTCCAAGAGGATTAAGGTTAAGTTGACTGTTCAAGCTGAGGTTCTTAATGGTGCTGTTCTTGAACAGTCTTATCCTGTTGAGTATACGGTTAGGGATAATCTGTGCGAGTCGTGTTCGAGGTTTCAGGCTAATCCTGATCAGTGGGTTGCTTCTATTCAGCTTAGGCAGCATGTTTCGCATAGGAGGACTTTCTTTTATCTCGAACAGTTGATTCTTAGGCACGATGCTGCTTCACGTGCCATTAGAATCCAGCAGGTGGATCAGGGGATTGATTTCTTCTTTGGGAATAAAAGTCATGCTAATAGCTTTGTGGAGTTTCTGAGGAAAGTTGTCCCCATTGAATACCGTCAGGACCAACAGCTGGTGTCTCATGATGTGAAAAGCAGTTTGTACAACTATAAGTACACTTACTCTGTTAAGATCTGTCCTGTTTGCCGTGAGGATCTTGTTTGCTTGCCTTCTAAAGTTGCTAGCGGCTTGGGGAACCTTGGTCCACTTGTGGTTTGCACAAAAGTCTCTGATAATATCACTCTACTTGATCCGAGAACTCTAAGGTGTGCCTTCTTGGATGCGAGACAGTACTGGAGGTCTGGGTTCCGATCTGCGCTCACCAGTAGACAACTTGTCAAGTACTTTGTGTTTGATGTTGAGCCACCTGTTGGTGAAGCGACTGTTGGTGGGCAAAAGTACGCTCTTTCCTATGTCCAGATTGCCCGTGAATCAGACATTGGTAAGATGTTTTATGTCCAAACTcTCTAGAgtcCGCAAATCACCAGTCTCTCTCTACAAATCTATCTCTCTCTATTTTCTCCAGAATAATGTGTGAGTAGTTCCCAGATAAGGGAATTAGGGTTCTTATAGGGTTTCGCTCATGTGTTGAGCATATAAGAAACCCTTAGTATGTATTTGTATTTGTAAAATACTTCTATCAATAAAATTTCTAATTCCTAAAACCAAAATCCAGTgacctgcag

***35S::EGFP-CRM1***

ctgcaggtcAACATGGTGGAGCACGACACTCTCGTCTACTCCAAGAATATCAAAGATACAGTCTCAGAAGACCAGAGGGCTATTGAGACTTTTCAACAAAGGGTAATATCGGGAAACCTCCTCGGATTCCATTGCCCAGCTATCTGTCACTTCATCGAAAGGACAGTAGAAAAGGAAGATGGCTTCTACAAATGCCATCATTGCGATAAAGGAAAGGCTATCGTTCAAGAATGCCTCTACCGACAGTGGTCCCAAAGATGGACCCCCACCCACGAGGAACATCGTGGAAAAAGAAGACGTTCCAACCACGTCTTCAAAGCAAGTGGATTGATGTGATAACATGGTGGAGCACGACACTCTCGTCTACTCCAAGAATATCAAAGATACAGTCTCAGAAGACCAGAGGGCTATTGAGACTTTTCAACAAAGGGTAATATCGGGAAACCTCCTCGGATTCCATTGCCCAGCTATCTGTCACTTCATCGAAAGGACAGTAGAAAAGGAAGATGGCTTCTACAAATGCCATCATTGCGATAAAGGAAAGGCTATCGTTCAAGAATGCCTCTACCGACAGTGGTCCCAAAGATGGACCCCCACCCACGAGGAACATCGTGGAAAAAGAAGACGTTCCAACCACGTCTTCAAAGCAAGTGGATTGATGTGATATCTCCACTGACGTAAGGGATGACGCACAATCCCACTATCCTTCGCAAGACCCTTCCTCTATATAAGGAAGTTCATTTCATTTGGAGAGGACctcgagaattcAACACAACATATACAAAACAAACGAATCTCAAGCAATCAAGCATTCTACTTCTATTGCAGCAATTTAAATCATTTCTTTTAAAGCAAAAGCAATTTTCTGAAAATTTTCACCATTTACGAACGATAGCCATGGCACTCATCTTTGGCACAGTCAACGCTAACATCCTGAAGGAAGTGTTCGGTGGAGCTCGGTACCCGGGGATCCATGGTGAGCAAGGGCGAGGAGCTGTTCACCGGGGTGGTGCCCATCCTGGTCGAGCTGGACGGCGACGTAAACGGCCACAAGTTCAGCGTGTCCGGCGAGGGCGAGGGCGATGCCACCTACGGCAAGCTGACCCTGAAGTTCATCTGCACCACCGGCAAGCTGCCCGTGCCCTGGCCCACCCTCGTGACCACCCTGACCTACGGCGTGCAGTGCTTCAGCCGCTACCCCGACCACATGAAGCAGCACGACTTCTTCAAGTCCGCCATGCCCGAAGGCTACGTCCAGGAGCGCACCATCTTCTTCAAGGACGACGGCAACTACAAGACCCGCGCCGAGGTGAAGTTCGAGGGCGACACCCTGGTGAACCGCATCGAGCTGAAGGGCATCGACTTCAAGGAGGACGGCAACATCCTGGGGCACAAGCTGGAGTACAACTACAACAGCCACAACGTCTATATCATGGCCGACAAGCAGAAGAACGGCATCAAGGTGAACTTCAAGATCCGCCACAACATCGAGGACGGCAGCGTGCAGCTCGCCGACCACTACCAGCAGAACACCCCCATCGGCGACGGCCCCGTGCTGCTGCCCGACAACCACTACCTGAGCACCCAGTCCGCCCTGAGCAAAGACCCCAACGAGAAGCGCGATCACATGGTCCTGCTGGAGTTCGTGACCGCCGCCGGGATCACTCTCGGCATGGACGAGCTGTACAAGATGGCGGCTGAGAAGTTAAGGGACTTGAGCCAGCCGATTGACGTCGGTGTGCTCGATGCCACTGTTGCGGCCTTCTTTGTTACCGGATCTAAAGAAGAGAGAGCTGCTGCGGACCAGATTTTGCGGGATTTGCAGGCTAATCCAGATATGTGGCTTCAAGTTGTCCACATTCTACAAAATACAAACAGCTTGGATACCAAGTTCTTTGCTCTGCAGGTTCTAGAAGGTGTTATAAAGTATAGATGGAATGCACTGCCTGTTGAACAACGAGATGGAATGAAAAATTACATCTCAGAGGTTATTGTACAGCTCTCGAGTAA TGAAGCATCTTTCAGATCAGAAAGGCTCTACTAAATGTCATTTTGGTCCAGATCGTGAAACATGATTGGCCGGCAAAGTGGACAAGCTTCATTCCTGATCTAGTTGCAGCTGCTAAAACTAGCGAAACTATCTGCGAAAATTGCATGGCCATTTTGAAACTCCTAAGTGAAGAGGTTTTTGATTTCTCAAGAGGAGAAATGACTCAGCAGAAGATTAAAGAGCTGAAACAATCTCTAAACAGTGAGTTTAAACTCATTCATGAGTTATGCCTATATGTCCTCTCAGCTTCTCAAAGACAGGATCTTATACGTGCAACACTGTCTGCATTGCATGCCTATCTTTCCTGGATTCCATTGGGTTACATTTTTGAGTCTACTTTGCTTGAGACCCTCCTTAAATTTTTTCCTGTGCCAGCATATAGGAACCTCACTATTCAATGTCTGACCGAGGTCGCAGCTCTTAATTTCGGGGACTTCTACAATGTTCAATATGTCAAAATGTATACCATATTTATAGGGCAGCTGCGGATAATTCTCCCACCGAGTACAAAGATCCCTGAGGCATATTCCAGTGGAAGTGGTGAAGAACAAGCATTTATCCAGAACCTGGCACTATTTTTCACTTCCTTTTTCAAGTTTCATATTCGAGTCCTAGAATCAACGCCAGAAGTTGTCTCTTTGTTACTCGCTGGTCTAGAATATCTCATTAATATATCTTATGTTGACGACACTGAAGTATTTAAGGTTTGTTTGGACTATTGGAACTCGTTGGTGTTGGAGCTATTTGATGCGCATCATAATTCTGATAACCCTGCAGTAAGTGCAAGCCTGATGGGTTTGCAGCCTTTCCTTCCTGGTATGGTTGATGGCCTTGGTTCTCAAGTCATGCAGCGGCGTCAACTTTATTCTCACCCAATGTCCAAATTAAGAGGGTTAATGA TTAACCGCATGGCGAAGCCTGAAGAAGTGCTTATTGTTGAAGATGAAAATGGGAACATCGTTCGTGAAACCATGAAGGACAATGATGTTCTTGTCCAATATAAGATAATGCGGGAGACATTAATCTACCTCTCACACCTTGACCATGATGATACCGAGAAGCAGATGTTGAGGAAGCTAAACAAACAATTAAGTGGGGAGGAATGGGCATGGAACAATTTGAACACTTTGTGCTGGGCTATTGGGTCTATTTCCGGATCTATGGCAGAAGATCAGGAAAACAGGTTTTTGGTGATGGTCATTCGTGATTTGTTGAATTTATGTGAAATTACCAAGGGAAAAGACAATAAAGCCGTTATTGCAAGCAACATCATGTATGTCGTTGGCCAGTATCCAAGATTCTTAAGGGCCCATTGGAAGT TTTTGAAGACAGTTGTGAACAAGTTGTTTGAATTCATGCATGAAACACATCCTGGTGTTCAGGACATGGCCTGTGATACATTCTTGAAAATAGTTCAAAAGTGCAAGCGAAAATTCGTTATTGTACAGGTTGGAGAGAATGAACCATTTGTATCTGAACTTCTAACAGGCCTTGCAACAACTGTTCAAGATCTTGAGCCTCATCAAATACACTCATTTTATGAATCAGTTGGTAATATGATCCAAGCAGAATCAGATCCTCAGAAGAGAGATGAATATCTCCAGAGGTTGATGGCACTCCCCAACCAGAAATGGGCAGAAATCATAGGACAGGCACGCCACAGTGTAGAATTCCTCAAGGATCAAGTTGTGATACGTACAGTGCTAAACATCCTACAGACTAATACTAGTGCTGCTACTTCACTGGGAACATACTTCTTATCCCAAATTTCCTTGATTTTCTTGGATATGTTGAATGTATACAGAATGTACAGTGAGCTTGTGTCAACCAACATTACTGAGGGAGGACCATATGCTTCCAAGACATCTTTTGTAAAACTCTTAAGATCGGTTAAGAGGGAAACACTTAAGCTGATAGAAACCTTTTTAGACAAAGCTGAAGACCAGCCACACATAGGGAAACAATTTGTGCCGCCAATGATGGAATCAGTACTTGGTGACTATGCGAGGAATGTGCCTGATGCTAGGGAATCCGAAGTTCTTTCACTCTTTGCAACGATTATAAACAAGTACAAGGCAACAATGTTAGACGACGTGCCTCACATATTTGAAGCTGTATTCCAGTGTACATTGGAGATGATAACTAAGAACTTTGAAGATTATCCAGAACACCGCCTCAAGTTTTTCTCATTACTCCGTGCTATTGCTACGTTTTGTTTCCCTGCCTTGATAAAGTTATCAAGTCCGCAACTGAAGCTAGTGATGGATTCAATTATCTGGGCATTTAGACATACTGAGAGAAATATTGCTGAAACCGGGCTTAATCTTTTGCTTGAGATGCTGAAAAACTTTCAGCAATCTGAATTTTGTAATCAATTCTACCGGTCATACTTTATGCAAATCGAGCAAGAAATATTTGCCGTTTTGACCGATACCTTCCATAAGCCTGGCTTCAAGCTACATGTGTTGGTGCTGCAGCAACTGTTTTGCCTGCCTGAGAGCGGTGCTTTGACAGAACCCTTGTGGGATGCTACAACCGTTCCTTACCCGTATCCGGACAACGTTGCATTTGTTCGCGAATACACCATTAAGCTACTGAGCTCTTCATTCCCAAACATGACTGCAGCAGAGGTCACACAATTTGTGAATGGACTATACGAGTCTAGAAATGACCCGTCTGGATTTAAGAATAACATTCGTGACTTCCTTGTACAGTCTAAGGAGTTTTCCGCTCAGGATAACAAAGATCTCTATGCTGAGGAAGCAGCTGCACAGAGAGAGAGAGAACGTCAAAGAATGCTTTCAATTCCTGGGCTTATTGCTCCTAATGAGATTCAAGACGAGATGGTGGACTCAGGATCCTCTAGAgtcCGCAAATCACCAGTCTCTCTCTACAAATCTATCTCTCTCTATTTTCTCCAGAATAATGTGTGAGTAGTTCCCAGATAAGGGAATTAGGGTTCTTATAGGGTTTCGCTCATGTGTTGAGCATATAAGAAACCCTTAGTATGTATTTGTATTTGTAAAATACTTCTATCAATAAAATTTCTAATTCCTAAAACCAAAATCCAGTgacctgcag


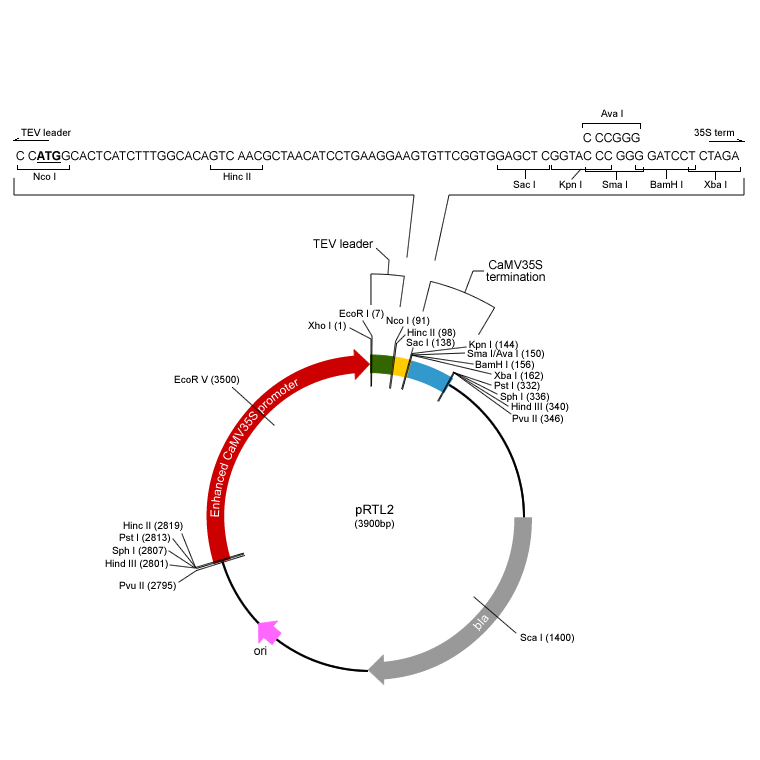


**C**

**Figure S4** **Plasmids construction for analysis of AtNMD3 shuttling between the nucleus and the cytoplasm**

**A.** Schematic diagrams of construct design of *EGFP-AtNMD3, EGFP-AtNMD3ΔNES* , *EGFP-AtNMD3ΔNLSΔNES,* and *EGFP-CRM1*

**B.** Sequences of the constructs: Green indicates *EGFP* CDS, Red indicates inserted CDS, Purple indicates enzymes position to insert into pRTL2 vector. Yellow indicates enzymes position to insert into pCambia 2300 vector

**C.** Scheme of pRTL2 plasmid
