# Supplementary material for: Arabidopsis NMD3 Is Required for Nuclear Export of 60S Ribosomal Subunits and Affects Secondary Cell Wall Thickening
Source: PLoS One. 2012 Apr 27;7(4):e35904. doi: 10.1371/journal.pone.0035904 (PMC3338764; doi:10.1371/journal.pone.0035904)
Supplement: Figure S5 — Yeast two-hybrid screening of proteins interacting with AtNMD3. (DOC) [file pone.0035904.s005.doc]

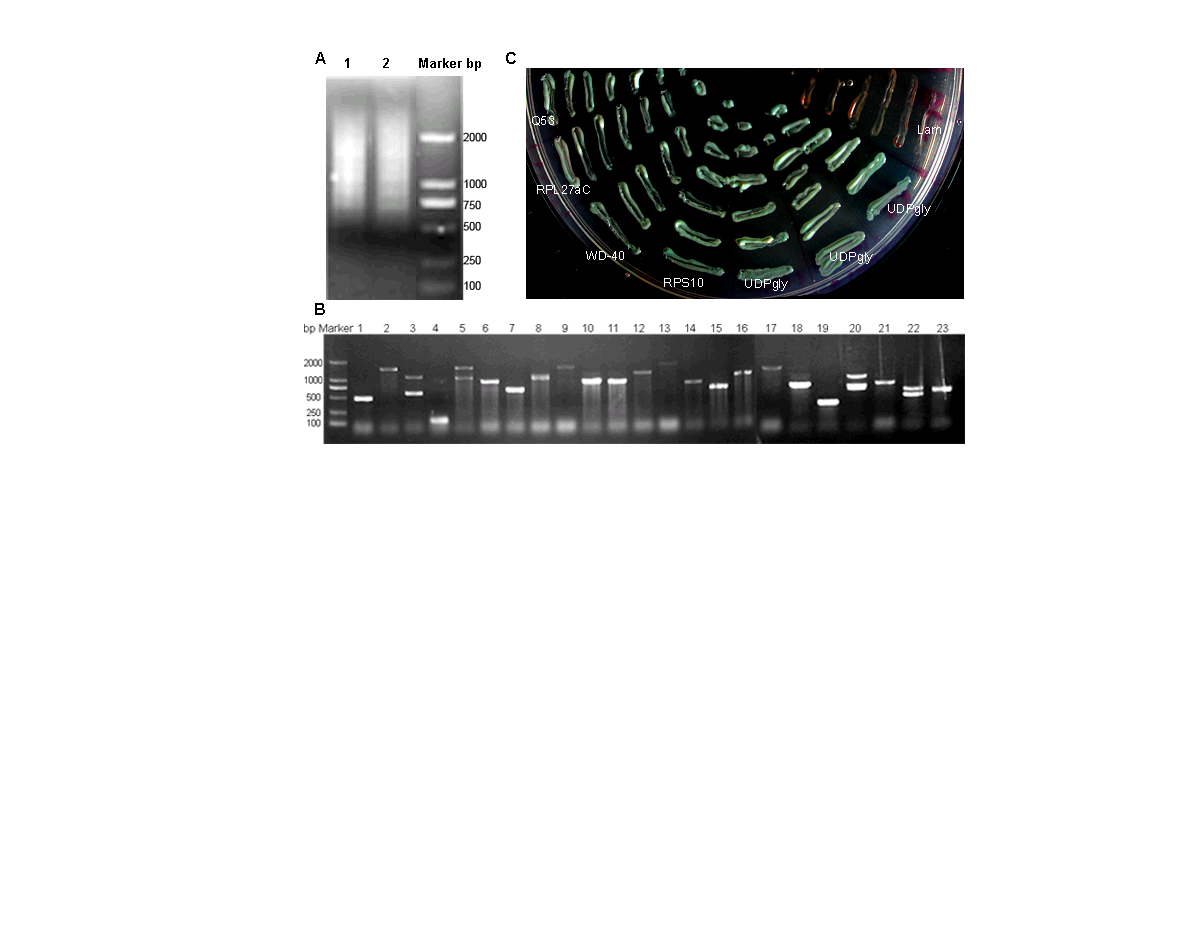


**Figure S5 Yeast two-hybrid screening of protein interacting with AtNMD3**

1. Examination of the quality of cDNA libraries, showing a good coverage of cDNAs reverse-transcribed from the equally mixed RNA extracted from the three types of plant materials (see the Brief procedure of yeast two hybrid screen). Lane 1 and 2 represented two biological replicates.
2. Examination of the insertion efficiency of the cDNA libraries, showing that inserts could be amplified from the all 23 randomly picked clones with a wide range of size from 250 bp to about 1500 bp.
3. Positive clones identified from the yeast two hybrid screening, which harbor genes potentially interact with AtNMD3. Note, the gene labeled RPL27a was now annotated as RPL15 (At1g70600. See [www.arabidopsis.org](http://www.arabidopsis.org/))

Brief procedure of yeast two hybrid screen:

Total RNA were isolated from 7-day-old,14-day-old seedlings and inflorescences by PolyAttract® mRNA isolation kits from Promega (cat#Z5310, USA).Constructing & Screening a yeast two hybrid Library were followed by BD Matchmaker Library construction Protocol A (PT3529-1,Clontech,US), briefly: synthesize first-strand cDNA using an Oligo (dT) Primer, Amplify double strand (ds) cDNA by Long Distance PCR, Purify ds cDNA with a BD CHROMA SPIN™ TE-400 Column **(**Clontech,US**)**. Library transformation efficiency is above 2.11X10^6^ transformants per 3ug pGADT7-Rec. Library titer is above 11.46X10^7^ clones/ml. After cotransforming yeast with pGADT7-Rec (Sma I-linearized) and ds cDNA. Transformants were selected on SD/–Leu plates. Randomly picked clones were screened for inserts by PCR. In this analysis, 23 out of 23 transformants carried plasmids with cDNA inserts. Note the wide range of insert sizes **(B)**. Transform competent yeast cells AH109 with the bait AtNMD3 and AD/ UDP-glycosyltransferase, RPL27ac(RPL15),RPS10,WD-40 plasmids,transfer well-isolated colonies to SD/–Ade/–His/–Leu/–Trp/X−α-Gal plates.Positive Control: AH109 (pGADT7-RecT+pGBKT7-53), Negative Control:AH109(pGADT7-RecT+pGBKT7-Lam), AH109(Bait,pGBKT7-AtNMD3) **(C)**
