# Supplementary material for: Arabidopsis NMD3 Is Required for Nuclear Export of 60S Ribosomal Subunits and Affects Secondary Cell Wall Thickening
Source: PLoS One. 2012 Apr 27;7(4):e35904. doi: 10.1371/journal.pone.0035904 (PMC3338764; doi:10.1371/journal.pone.0035904)
Supplement: Figure S6 — Sequence similarity of AtRPL15 with yeast RPL28A and a computational model of RPL28A localization in the ribosome. (DOC) [file pone.0035904.s006.doc]

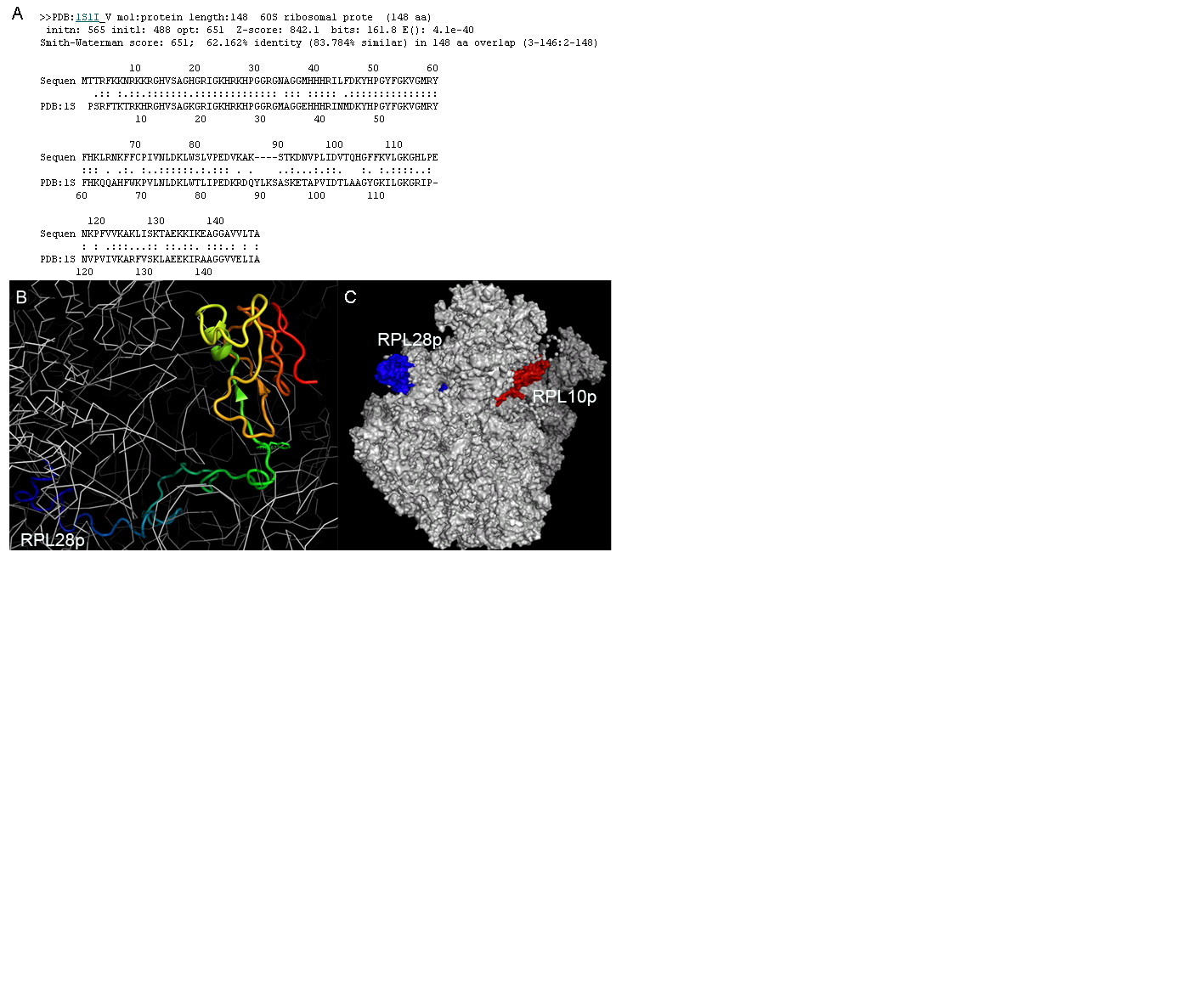


**Figure S6 Sequence similarity of AtRPL15 with yeast RPL28A and a computational model of RPL28A localization in the ribosome**

1. Alignment of AtRPL15 and RPL28 (Yeast) by Fasta33 (<http://www.ebi.ac.uk/Tools/sss/fasta/>) resulting in 62.16% in 148 aa overlap. AtRPL15 (previously annotated as RPL27aC) has highest sequence similarity to yeast RPL28. AtRPL15 has also been annotated as L27A, L29, YL24, and RP62.
2. Computational predicted structure of RPL28 by pymol (<http://www.pymol.org/>) (colored by blue at the N-terminal red at the C-terminal). The first 65 amino acid residues in N-terminal are inserted to the 60S subunit of Ribosome.
3. Computational predicted surface of yeast 80S Ribosome by pymol (<http://www.pymol.org/>). RPL28 (blue) was located opposite to RPL10 (red) in the complex.
