# Supplementary material for: Arabidopsis NMD3 Is Required for Nuclear Export of 60S Ribosomal Subunits and Affects Secondary Cell Wall Thickening
Source: PLoS One. 2012 Apr 27;7(4):e35904. doi: 10.1371/journal.pone.0035904 (PMC3338764; doi:10.1371/journal.pone.0035904)
Supplement: Figure S7 — AtNMD3 does not complement the yeast temperature sensitive nmd3 mutant. (DOC) [file pone.0035904.s007.doc]

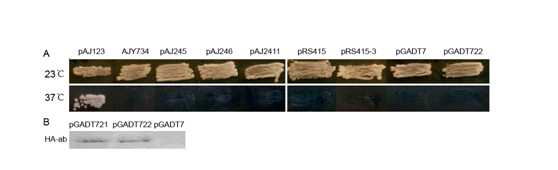


**Figure S7 AtNMD3 does not complement the yeast temperature sensitive *nmd3* mutant**

**A.** AtNMD3 failed to complement yeast NMD3p temperature sensitive mutant AJY734 (MATa ade2 ade3 leu2 lys2-801 ura3-52 nmd3-4). Plasmids: pAJ2411 [LEU2-CEN (YpNMD3-eGFP-AtNMD3)], pAJ246 [LEU2-CEN (YpNMD3-AtNMD3)], pRS415-3 [pRS415-AtNMD3 (GDP CEN,LEU2 GDP)] with weak promoter. pADT7-21,22 [pADH-HA-AtNMD3] with strong promoter. Positive control: pAJ123 [LEU2-CEN (YpNMD3-NMD3p)]; Negative control: pAJ245 [LEU2-CEN (YpNMD3)], pAJ415 [LEU2-CEN], and pGADT7

**B.** Immunoblot detection AtNMD3 expression in AtNMD3 transformed AJY734 *nmd3-4* mutant, demonstrating that AtNMD3 has been transformed into and expressed in the transgenic yeast cells.
