# Supplementary material for: Arabidopsis NMD3 Is Required for Nuclear Export of 60S Ribosomal Subunits and Affects Secondary Cell Wall Thickening
Source: PLoS One. 2012 Apr 27;7(4):e35904. doi: 10.1371/journal.pone.0035904 (PMC3338764; doi:10.1371/journal.pone.0035904)
Supplement: Figure S8 — Construction of the transgenic Arabidopsis overexpressing truncated AtNMD3 without a NES (the AtNMD3ΔNES OE line). (DOC) [file pone.0035904.s008.doc]

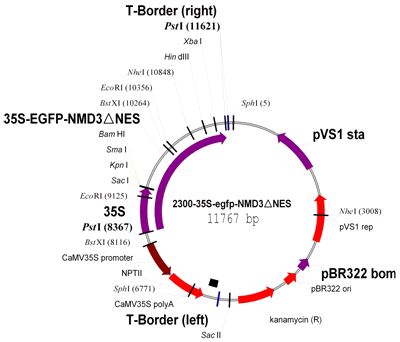

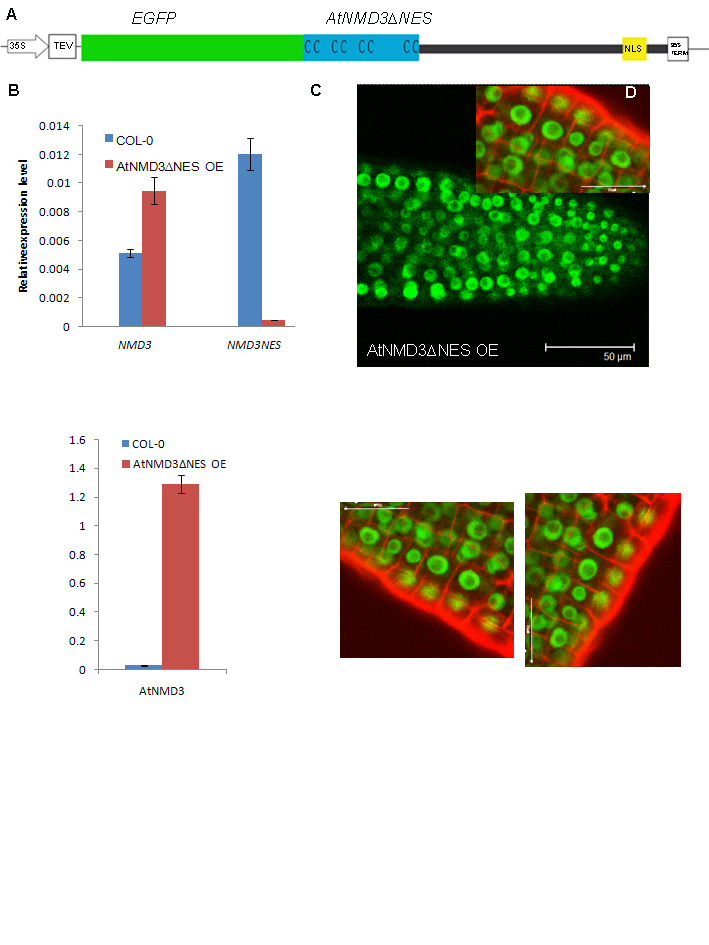


**B**


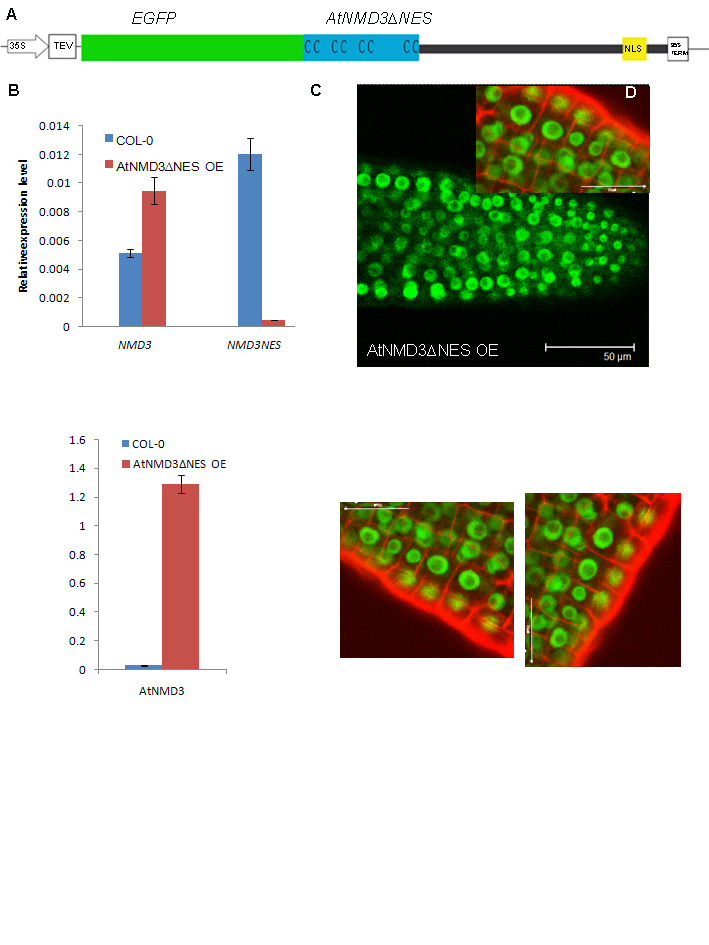


**C**

**D**

**E**

**Figure S8 Construction of the transgenic Arabidopsis overexpressing truncated AtNMD3 without NES (the AtNMD3ΔNES OE line)**

1. Schematic diagram of AtNMD3ΔNES OE vector and the detailed information the plasmid structure of pCAMBIA2300.
2. Detection of insertion sites of transgenic Arabidopsis overexpressing the *AtNMD3ΔNES* construct in different independent transgenic lines (blue arrows pointed, Chr1: 8882668 and Chr4: 11763249)
3. Detection of efficiency of dominant negative suppression of *AtNMD3* in the *AtNMD3ΔNES OE* transgenic line: expression levels detected using primers for NMD3 (sequence shared by endogenous *AtNMD3* and exogenous *AtNMD3ΔNES* genes) was increased as expected (left pair); while that using primers for s endogenous *AtNMD3* (cover the *NES* sequence) was dramatically decreased, indicating a successful dominant negative suppression of *AtNMD3*.

**D** and **E.** EGFP-AtNMD3ΔNES protein was strongly trapped in the nuclei in the *AtNMD3ΔNES OE* transgenic line, consistent with the observation in transient expression of EGFP-AtNMD3ΔNES in the protoplasm shown in Figure 2 (**D**). The nucleus localization was further verified in (**E)**，in which cell wall were stained as red with Propidium Iodide.
