# Supplementary material for: Arabidopsis NMD3 Is Required for Nuclear Export of 60S Ribosomal Subunits and Affects Secondary Cell Wall Thickening
Source: PLoS One. 2012 Apr 27;7(4):e35904. doi: 10.1371/journal.pone.0035904 (PMC3338764; doi:10.1371/journal.pone.0035904)
Supplement: Figure S9 — Examination of AtNMD3 expression in the commercially obtained atnmd3 mutants. (DOC) [file pone.0035904.s009.doc]

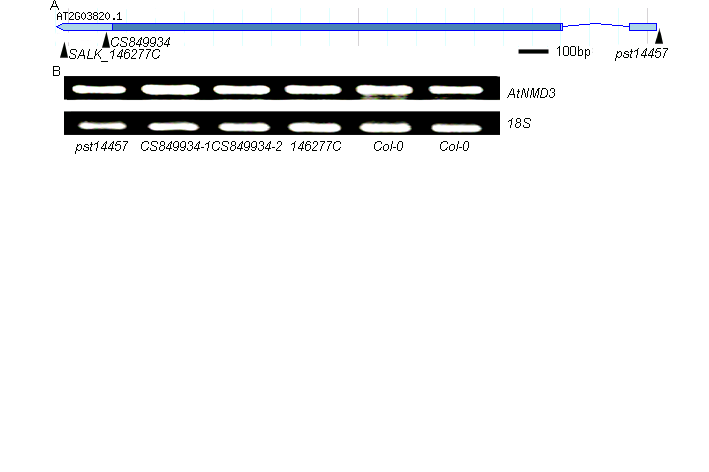


**Figure S9 Examination of AtNMD3 expression in the commercially obtained *atnmd3* mutants**

1. Schematic diagram of *atnmd3* mutants T-DNA insertion according to information provided by stock resources (see Materials and Methods).
2. No reduction of *AtNMD3* expression was detected in the all three *atnmd3* mutants by RT-PCR
