# Supplementary material for: Arabidopsis NMD3 Is Required for Nuclear Export of 60S Ribosomal Subunits and Affects Secondary Cell Wall Thickening
Source: PLoS One. 2012 Apr 27;7(4):e35904. doi: 10.1371/journal.pone.0035904 (PMC3338764; doi:10.1371/journal.pone.0035904)
Supplement: Figure S10 — Detailed quantitative data for dynamic changes of RER/SER during stem development in the wild-type and AtNMD3-ΔNES OE lines. (DOC) [file pone.0035904.s010.doc]

**A B**

**Figure S10 Detailed quantitative data for dynamic changes of RER/SER during stem development in the wild type and AtNMD3-ΔNES OE line**

1. Transmission Electron Microscopy of the subcellular structures of a sample collected from position 2 in wild type (Col-2) stem (detailed description of sampling process see Figure 7 and text in the “result” session). Arrows show rough ER (RER). (Bar = 500nm)
2. Transmission Electron Microscopy of the the subcellular structures of a sample collected from position 3 in AtNMD3-ΔNES OE line stem (detailed description of sampling process see Figure 7 and text in the “result” session). (Bar = 500nm)

**Detailed data for the quantitative analysis* of dynamic changes of RER/SER see Supplementary Table S2**

* Lengths of RER or SER (Arrows indicated structures) were measured using ImageJ (NIH,USA) on the selected representative TEM images. The observed cytoplasm area containing the measured RER and/or SER counted using ImageJ were as a unit (the area of plasmids and mitochondria in the counted area were excluded). At least 10 transgenic plants with the most severe phenotypes in their third generation were examined for the wall thickness of fibers, rough endoplasmic reticulum (RER) and smooth endoplasmic reticulum (SER)
