# Supplementary material for: Arabidopsis NMD3 Is Required for Nuclear Export of 60S Ribosomal Subunits and Affects Secondary Cell Wall Thickening
Source: PLoS One. 2012 Apr 27;7(4):e35904. doi: 10.1371/journal.pone.0035904 (PMC3338764; doi:10.1371/journal.pone.0035904)
Supplement: Figure S11 — Examination of selected RPL gene expression during stem development in the wild type and AtNMD3-ΔNES OE line. (DOC) [file pone.0035904.s011.doc]

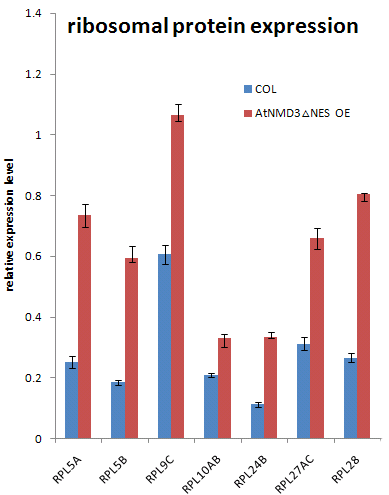


**Figure S11 Examination of selected *RPL* gene expression during stem development in the wild type and AtNMD3-ΔNES OE line**

Expression levels of selected ribosomal protein genes detected by qPCR were upregulated in the AtNMD3ΔNES OE line. Primers used for PCR analysis were listed in Table S1. UBIQUITIN10 (UBQ10) was used as reference.
