# Supplementary material for: Arabidopsis NMD3 Is Required for Nuclear Export of 60S Ribosomal Subunits and Affects Secondary Cell Wall Thickening
Source: PLoS One. 2012 Apr 27;7(4):e35904. doi: 10.1371/journal.pone.0035904 (PMC3338764; doi:10.1371/journal.pone.0035904)
Supplement: Figure S12 — Immunoblot detection of antibody against AtNMD3 and RPL15. (DOC) [file pone.0035904.s012.doc]

**A Anti-AtNMD3 B Anti-RPL15**


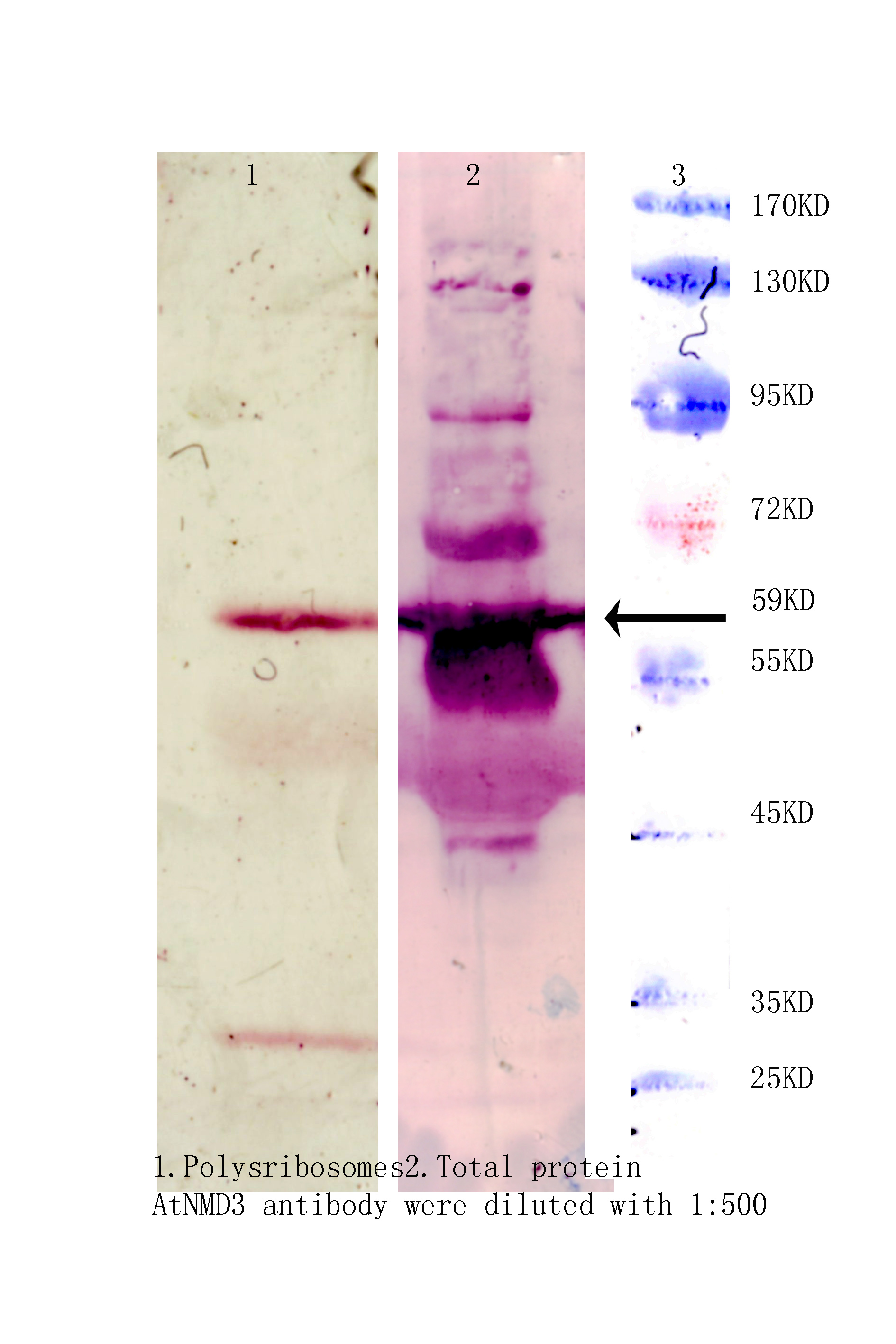

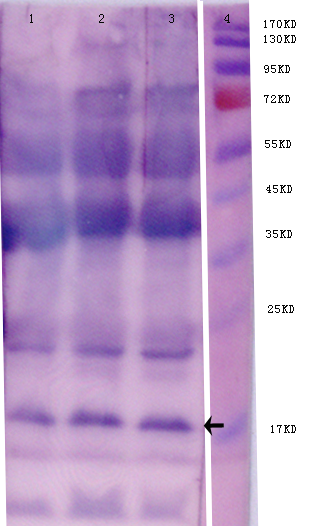


**Figure S12 Immunoblot detection of antibodies against AtNMD3 and RPL15**

1. Detection of AtNMD3 protein by the antibody against AtNMD3 (diluted by 1:500 ratios) in the polysome extracted from wild type 14-day- old seedlings (Lane 1, 15 µl sample solution) and in total protein extracted from wild type 14-day- old seedlings (Lane 2, 15 µl sample solution). Arrow indicates the position of the protein band recognized by the AtNMD3 antibody. Lane 3 is the protein markers for molecular weight.
2. Detection of RPL15 protein by the antibody against RPL15 (diluted by 1:200 ratios) in the total protein extracted from wild type 14-day- old seedlings (Lane 1: 10 µl sample solution; Lane 2: 15µl sample solution), and that from YFP-RPL28 14-day- old seedlings (Lane 3: 15µl sample solution). Arrow indicates the position of the protein bands with predicted molecular weight of RPL15. Lane 4 is the protein markers for molecular weight.
