# Supplementary material for: Arabidopsis NMD3 Is Required for Nuclear Export of 60S Ribosomal Subunits and Affects Secondary Cell Wall Thickening
Source: PLoS One. 2012 Apr 27;7(4):e35904. doi: 10.1371/journal.pone.0035904 (PMC3338764; doi:10.1371/journal.pone.0035904)
Supplement: Table S1 — Oligonucleotides used in this study. (DOC) [file pone.0035904.s013.doc]

**Table S1 Oligonucleotides used in this study**

| **Primers for plasmid construction** | | |
| --- | --- | --- |
| Primers | Enzyme | Sequences |
| pRTL2-NMD3-5 | BamHI | 5 CGGGATCCatgtcagtaatggatgaatcaggc |
| pRTL2-NMD3-450 | XbaI | 5 GCTCTAGAgaaactcttcgtattctttgtctgt |
| pRTL2-NMD3-360 | XbaI | 5 GCTCTAGAgagtttggacataaaacatcttacc |
| pRTL2-NMD3-R | XbaI | 5 GCTCTAGAgttcagcagccatgtcgtcctcgtca |
| GFP-NcoI | NcoI | 5 TATCCATGGtgagcaagggcgaggagct |
| GFP-CRM1a R |  | 5 Cttct cagccgccatCTTGTACAGCTCGTCC |
| CRM1a-F-BamH | BamHI | 5 CGGGATCCatggcggctgagaagttaagggact |
| GFP-R-BamH | BamHI | 5 CGGGATCCcttgtacagctcgtccatgccgaga |
| CRM1a-GFPF2 |  | 5 GGACGAGCTGTACAAGATGGCGGCTGAGAAGTT |
| CRM1aR | BamHI | 5 CGGGATCCtgagtccaccatctcgtcttgaatc |
| GFP NMD3 R |  | 5 CATCCATTACTGACATCTTGTACAGCTCGTCC |
| NMD3 GFP F2 |  | 5 GGACGAGCTGTACAAGATGTCAGTAATGGATG |
| NMD3-RNAi-SenseF | XhoI | 5 CCGCTCGAGGGCAGCATGTTTCGCATAGGAGG |
| NMD3-RNAi-SenseR | SpeI | 5 GGACTAGTCAACAGGTGGCTCAACATCAAAC |
| NMD3-RNAi-AntiF | BamHI | 5 CGCGGATCCCAACAGGTGGCTCAACATCAAAC |
| NMD3-RNAi-AntiR | XbaI | 5 GCTCTAGAGGCAGCATGTTTCGCATAGGAGG |
|  |  |  |
| **Primers for qPCR** | | |
| Gene | AGI code | Sequence |
| AtNMD3 | At2g03820 | NMD415-F5' GTTAGGGATAATCTGTGCGAGTC 3' |
|  |  | NMD521-R5' AAGAAAGTCCTCCTATGCGAAAC 3' |
|  |  | NMD3NESF5'CTTTGAGGAAGAAGACGATGATGA 3' |
|  |  | NMD3NESR5'TGAGAGAAACACAAGAGAAGCAAA3' |
| *4CL1F* | At1g51680 | TACTGATGTTGCTGTTGTCGCAAT |
| *4CL1R* |  | GGAACTTCACCAGCTGCTTCTTCT |
| *4CL2F* | At3g21240 | TGCTAAGTTTCCTAACGCCAAGC |
| *4CL2R* |  | GCTTCTGTCATCCCATAGCCCT |
| *AtHB8Rf1* | At4g32880 | TGTGTTGCTCACTCAAGGCCTTA |
| *AtHB8Rr1* |  | TGTCGAAGATCTTGTCGAGAGTGA |
| *AtMYB26 FP* | At3g13890 | CCA TGG ATG TTG GAG CTC TGT T |
| *AtMYB26 RP* |  | GCT TCC ACG TTT AAG ATG CAG GTC T |
| *BFN1RTf1* | At1g11190 | ATCGCTTGTCCACACAAGTATGC |
| *BFN1RTr1* |  | TTCACCAGACTTGACGCCTTTGTA |
| *C4HF* | At2g30490 | TCGCCGCGATTGAGACAAC |
| *C4HR* |  | GCTCTGCAATTCCCCACTCG |
| *CAD4F* | At3g19450 | ATCCTATGGTTCCTGGGCACGA |
| *CAD4R* |  | TCTCCTACGGTGAACTTGCTCACA |
| *CCR1F* | At1g15950 | GACCCTGAGGCTGTCGTTGA |
| *CCR1R* |  | GCCGTAACAATACCAATTCTTGGT |
| *CCR2F* | At1g80820 | GAGGTTGTTGAGATTCTGGCCA |
| *CCR2R* |  | TTCTCGTCCGAACACTTGGTG |
| *CESA1F* | At4g32410 | GAGCTGAGATGGAGCGGTGT |
| *CESA1R* |  | CTGCTCGTTCCTCCACCAAT |
| *COMT1F* | At5g54160 | ATCTCCCACATGTCATCGAAGA |
| *COMT1R* |  | TCTCCTCCAACATGCTCAATACC |
| *CslC8F* | At2g24630 | GAGCTGGGTTGGATCTATGGC |
| *CslC8R* |  | AATGCATCTTGAATCCCGTCA |
| *CslD5F* | At1g02730 | AGCTATTTGCCTTCTTACCGGC |
| *CslD5R* |  | CATGCTTGCTAGGTTTGATAGCG |
| *F5HF* | At5g04330 | CATGGATGTGATGTTCGGAGG |
| *F5HR* |  | GGTTAGCACCCATTCGATTGC |
| *GAUT1F* | At3g61130 | CGAATGCACAACAGAGGATGG |
| *GAUT1R* |  | CACGGAAAGCGTCTCTTTTTG |
| *HCTF* | At5g48930 | TTCCCGAAGTTGATCACTCCG |
| *HCTR* |  | GTCACCTGCAAAACGAGAAGC |
| *IRX10F* | At5g61840 | CATATGCTCCACCACAGAAGATGC |
| *IRX10R* |  | CAGCATGGCTTGTTTCATCG |
| *IRX12F* | At2g38080 | GGTGGATGGGTCGTCATGAGATTC |
| *IRX12R* |  | CGTGGCGTGATGTTGATATGTCGCCC |
| *IRX3F2* | At5g17420 | GGCAAACTCAAGTGGCTTGAGCG |
| *IRX3R2* |  | TAACTCCGCTCCATCTCAATTCC |
| *IRX4RTf* | At1g15950 | CGTTATCTCCTAGCCGAGAGTGCTC |
| *IRX4RTr* |  | TGCCATTTTCCACGGATTCTTGCGATGC |
| *IRX5Rf1* | At5g44030 | CGGCAAGTTCATCATTCCTACGA |
| *IRX5Rr1* |  | CACTCCACCGGAGTTCTAAGA |
| *IRX8F* | At5g54690 | TGGCAAAATCTGAACGAGAACA |
| *IRX8R* |  | TCAGTCCCGGTGGTAACGTC |
| *IRX9F* | At2g37090 | TCATTCTCTCCAAAGCTGGTCAT |
| *IRX9R* |  | CGGTAACGTCGCTTAGGAATAGA |
| *MYB58F* | At1g16490 | CCAGAGAACAGAGCTCTTCAAGAG |
| *MYB58R* |  | ATGTATGAGGAGCGTAACTCTC |
| *MYB63F* | At1g79180 | GAACAGCTCAGGCTCAAGAGCAAC |
| *MYB63R* |  | ATGTATCATGAGCTCGTAGTTCTT |
| *NST1F:* | At2g46770 | TCATCCGACCGAGGAAGAGC |
| *NST1R:* |  | GAAGCTCCTCCGACGGGACT |
| *NST2F:* | At3g61910 | TCACCCAACCGAGGAAGAGC |
| *NST2R:* |  | CATGATCGCCACACGAGGAG |
| *NST3F:* | At1g32770 | GCCCACCTTTCACAACACTCA |
| *NST3R:* |  | GCTTGGAGTCAGGAAACTTTGGTC |
| *PARVUSF* | At1g19300 | GTACACGTCACGCGAAGAG |
| *PARVUSR* |  | AGAATCCAACGAGAACGGCGTTTG |
| *VND6F* | At5g62380 | CCCAACTACAATAATGCAAC |
| *VND6R* |  | GCTCATGATTAGCTGAGAA |
| *VND7F* | At1g71930 | GGACGAATAAAGATCAGAACGA- |
| *VND7R* |  | ATGCGGATGTATGACTTGTGTC |
| *XCP1F* | At4g35350 | GAGGCTTCAGGAAGAGACTTCCAG |
| *XCP1R* |  | CACTTGGTCTTGGTAGGATATGAGG |
| *RPL10AB-F* | At2g27530 | GAGGAGGCAGAGAAAATGGGAC |
| *RPL10AB-R* |  | TCACCTTTGCTTCCAATGACTCTT |
| *RPL10c657* | At1g66580 | CATTGGCTAACCGTCAACCTG |
| *RPL10c778* |  | TGAAACAGAAAAGAGCAGAAACTACA |
| *RPL24B-F* | At3g53020 | GGAAAGAATCAAAAAGACCAAAGA |
| *RPL24B-R* |  | CAAAAGTGAGAGACAAAAACGAAA |
| *RPL153481* | At1g70600 | GCACAACAGCACCACCAGCT |
| *RPL155311* |  | AGGCGAAATCAACCAAGGACA |
| *RPL28A-F* | At2g19730 | AAGGAGACTAACAACCTCACCAAC |
| *RPL28A-R* |  | GAGGATAGACTTGTTGACAGAGAGC |
| *RPL5A-F* | At3g25520 | AATGGATGACGAGTATGAGGGAAA |
| *RPL5A-R* |  | TTTCCTGTTGTGGTCCTGATAAGT |
| *RPL5B-F* | At5g39740 | AAGGAGAGATGGGAAGACTGATTA |
| *RPL5B-R* |  | GGTAAGACCAACAGTGAGTCCATA |
| *RPL9C-F* | At1g33140 | GAGGTTTCCGTTACAAGATGAGGT |
| *RPL9C-R* |  | ATCCAACATCTCTACCTTCCTCAC |
